# Hapten-Induced Contact Hypersensitivity, Autoimmune Reactions, and Tumor Regression: Plausibility of Mediating Antitumor Immunity

**DOI:** 10.1155/2014/175265

**Published:** 2014-05-15

**Authors:** Dan A. Erkes, Senthamil R. Selvan

**Affiliations:** ^1^Immunology and Microbial Pathogenesis Graduate Program, Thomas Jefferson University, Philadelphia, PA 19107, USA; ^2^Division of Solid Tumor, Department of Medical Oncology, Thomas Jefferson University, Curtis Building, Suite 1024B, 1015 Walnut Street, Philadelphia, PA 19107, USA

## Abstract

Haptens are small molecule irritants that bind to proteins and elicit an immune response. Haptens have been commonly used to study allergic contact dermatitis (ACD) using animal contact hypersensitivity (CHS) models. However, extensive research into contact hypersensitivity has offered a confusing and intriguing mechanism of allergic reactions occurring in the skin. The abilities of haptens to induce such reactions have been frequently utilized to study the mechanisms of inflammatory bowel disease (IBD) to induce autoimmune-like responses such as autoimmune hemolytic anemia and to elicit viral wart and tumor regression. Hapten-induced tumor regression has been studied since the mid-1900s and relies on four major concepts: (1) *ex vivo* haptenation, (2) *in situ* haptenation, (3) epifocal hapten application, and (4) antigen-hapten conjugate injection. Each of these approaches elicits unique responses in mice and humans. The present review attempts to provide a critical appraisal of the hapten-mediated tumor treatments and offers insights for future development of the field.

## 1. Introduction

Haptens are small molecules that elicit an immune response when bound to a carrier protein [1]. Haptens have been used to boost immune responses to antigens, to study ACD and IBD, and to induce autoimmune responses, viral wart regression, and even antitumor immunity. For years, haptenated protein (bovine serum albumin (BSA) or ovalbumin (OVA)) was mainly utilized to induce strong immune responses in animal models to help unravel the basics of T- and B-cell-mediated responses. Paul et al. [2] immunized BSA-tolerized rabbits with DNP-modified BSA producing antibodies to the dinitrophenyl (DNP)-BSA conjugate, BSA alone, and DNP alone, suggesting potential cross-reactive responses. Classically, B-cells are known to recognize the DNP-BSA conjugates* via* membrane bound IgM, process them, make antibody against the DNP, and present the BSA to CD4+ T-cells. These abilities of haptens have made them a tantalizing molecule for use in several settings. Haptens have been widely used to induce CHS, the animal model of ACD, a type IV delayed hypersensitivity reaction that is one of the most prevalent skin diseases in the world [3, 4]. CHS has two phases, a “sensitization” phase where the hapten is applied to skin for the first time, followed by an “elicitation” phase where the hapten is applied to a different skin area of the animal [3–5]. An in-depth analysis of the innate and adaptive immunologic mechanisms of CHS and ACD is covered in three recent reviews by Martin et al. [6], Christensen and Haase [5], and Honda et al. [4]. In this review, we will briefly cover these immune reactions to allow for a general understanding of how these reactions may apply to antitumor immunity.

Some hapten-mediated responses are correlated to drug-induced autoimmune reactions. When a drug is metabolized, its metabolites can form potent haptens, which bind self-protein and sometimes elicit autoimmune responses [7, 8]. Hapten-carrier conjugates have been used in the past as drug-abuse therapies [9, 10], inducing an immune response against the drug of interest. Haptens have also been used to create autoimmune models in mice, such as IBD [11–17], and to cause viral wart regression* via* epifocal hapten application [18, 19]. The ability of haptens to cause autoimmunity and wart regression is an important concept to consider when applying the use of haptens to cancer immunotherapy setting, as the immune response to cancer is similar to an autoimmune response [20]. Indeed, haptens have been tested as a treatment of cancer several times in the past. In this review, we examine the four main concepts of hapten-mediated antitumor treatment: (1)* ex vivo* haptenation [21–31], (2)* in situ* haptenation [32, 33], (3) epifocal hapten application [34–42], and (4) antigen-hapten administration [43–47]. Despite the wealth of experiments in this field, the mechanisms underlying these treatment approaches are largely unclear and require further study. We attempt to give a critical analysis of the use of haptens to induce tumor regression and suggest studies that must be done to fill the large knowledge gaps and further the field.

## 2. Haptens and Contact Hypersensitivity

Haptens are <1 kDa in size and elicit an immune response when bound to a carrier protein, including tolerized antigen. Haptens are not immunogenic by themselves, as they are too small to be recognized by the immune system. Most haptens are electrophilic compounds that covalently bind to nucleophilic residues creating new antigenic epitopes; an exception to this would be metal ions functioning as haptens [1]. Most haptens act as cutaneous allergens, eliciting ACD-like reaction on the skin. The most common haptens are urushiol (the toxin in poison ivy), fluorescein, nickel, oxazolone (Ox), DNP, and phosphorylcholine. Each hapten has a unique property that determines its allergenicity in terms of how quickly the hapten binds, how readily it can permeate the skin, and its electrophilicity, hydrophobicity, and subsequent bioavailability [1]. Varying mouse strains also greatly affect the immune stimulatory ability of the hapten. Contact hypersensitivity is usually measured through ear swelling, as the secondary challenge application is on the ear (elicitation phase); this is the widely used method to confirm sensitization of a hapten and unravel the immune mechanisms of haptens and CHS [3]. The body of literature on haptens and CHS reveals the use of several different animal models and haptens that lead to conflicting explanations of a certain step in the immune pathology of CHS, which should be considered when creating a general mechanism of CHS. While outlining our understanding of the mechanisms of CHS, we primarily focus on the aspects that will be important for hapten-mediated tumor regression.

### 2.1. The Sensitization Phase of Contact Hypersensitivity

**Figure 1:**
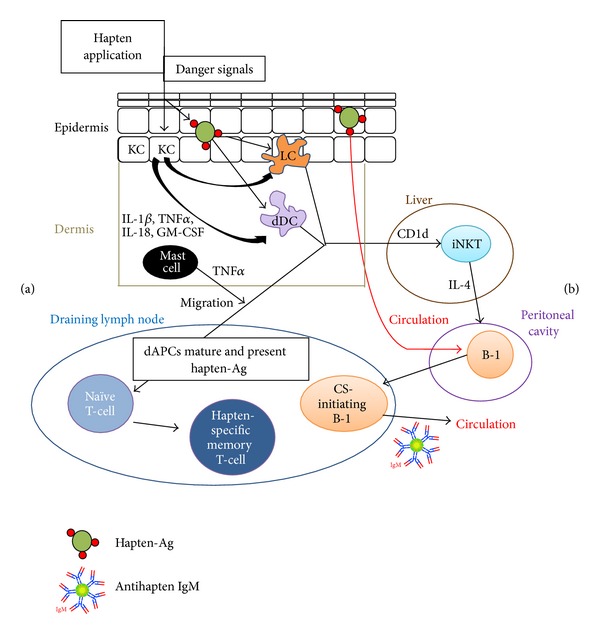
The likely pathway of the “sensitization” phase of contact hypersensitivity. (a) Hapten application induces strong innate immune mechanisms, causing cell death and the release of danger signals and endogenous ligands, leading to cytokine release, IL-1*β*, IL-18, TNF*α*, and GM-CSF, by keratinocytes (KC). This release will stimulate dermal antigen-presenting cells (dAPCs), langerhans cells, and dermal dendritic cells, to take up haptenated antigen and migrate to the dLN to activate naïve T-cells. Mast cells will aid in this migration by releasing TNF*α*. (b) iNKT cells in the liver will be activated by APCs presenting haptenated glycolipid by CD1d. This will cause cytokine release, IL-4, to stimulate naïve B-1 cells in the peritoneal cavity, along with the binding of hapten-antigen by membrane IgM. This will cause migration of these cells to the dLN, and subsequent maturation into CS-initiating B-1 cells, which release antihapten IgM into circulation.

The sensitization phase is when a hapten is first applied to the skin of an animal, typically the shaved abdomen, to prime the immune system toward the hapten.  Figure 1 summarizes some of the cells and mechanisms thought to be involved in this priming event. Upon application to the skin, haptens immediately interact with keratinocytes (KC), langerhans cells (LC), and dermal dendritic cells (dDC). Hapten binding to KCs causes them to release IL-1*β*, IL-18, TNF*α*, and GM-CSF, activating LCs and dDCs and inducing their migration to the draining lymph node where they mature and present hapten-antigen to naïve T-cells [4–6, 48–52]. Dinitrofluorobenzene (DNFB) application to dermal dendritic cells* in vitro* upregulates MAPK and CD40, a coactivation signal for antigen-presenting cells (APCs) and T-cells [53]. Haptenation also causes the release of “danger signals,” such as hyaluronic acid (HA), extracellular matrix ligands for Toll-like receptors, such as extradomain A+ fibronectin containing extra type III domain A (FnEDA+), prostaglandin E2 (PGE2), reactive oxygen species (ROS), heparin sulfate, tenascin, B defensins, and fibrinogen [4, 5, 54], from haptenated cells, which play an integral role in innate immune activation [6]. For instance, blocking HA degradation significantly reduces CHS sensitization [6], while the release of PGE2 activates LCs and induces their migration [55]. The* in vitro* formation of ROS in DCs is thought to cause degradation of the extracellular matrix, creating endogenous ligands for toll like receptors (TLRs)-2 and -4, as well as nucleotide-binding oligomerization domain (NOD) like receptors (NLRs) [4, 6]. Keratinocytes are mainly stimulated by NLR-dependent mechanisms, specifically NLR family, pyrin domain containing 3 (NLRP3) [6]. NLRP3 stimulation triggers caspase-1 activation, which causes the release of IL-1*β* and IL-18 from keratinocytes and APCs. This NLR-dependent pathway is stimulated by adenosine triphosphate (ATP) efflux from haptenated and subsequently damaged cells. ATP binds to the purinergic receptor, P2RX7, a ligand gated ion channel that allows the release of K+ from the cell and provides further innate activation signals for LCs and dDCs, helping them mature [6].

Langerhans cells play a pivotal but controversial role in the sensitization phase; when LCs are depleted, the ear-swelling responses are reduced [50]. Further, LCs and dDCs work together to initiate CHS sensitization [56, 57]. The role of the LCs seem to be area and time of depletion dependent, for instance, LCs had a larger role in the flank than in the ear and LC depletion three days prior to hapten priming did not impair CHS but LC depletion 1 day prior did impair CHS [58, 59]. It was shown that only dDCs, not LCs, migrate to the draining lymph node (dLN) to activate and stimulate hapten-specific T-cells [52, 60]. Despite this controversy, LCs cells have been shown to play an important role in CHS sensitization.

Mast cells are also thought to play a role in CHS sensitization. Initial reports using mast cell deficient mice through a c-Kit mutation showed that CHS was enhanced, although this is hard to interpret as c-Kit mutation affects many cells [4, 60]. Diphtheria toxin-induced mast cell-deficient mice had reduced CHS and T-cell priming [4, 61, 62]. Mast cells also stimulate dDCs via intercellular adhesion molecule-1 (ICAM-1) or leukocyte function-associated antigen-1 (LFA-1) and TNF*α* [4, 61, 62]. Mast cells and dendritic cells are critical during the sensitization phase, causing DC migration and maturation [4, 5, 61, 62].

Upon maturation by Keratinocyte stimulation, langerhans cells and dDCs migrate to the dLN. The dermal APCs activate naive T-cells and invariant natural killer T (iNKT) cells by presenting the haptenated antigen (peptide and lipid) via MHCI/II or CD1d, respectively. Peptide presentation depends on whether the haptenated protein becomes internalized and processed via the endosomal compartments, followed by MHC-I presentation [63], or whether the haptenated proteins are on the extracellular surface and cross presented via MHC-I to CD8-T-cells [64]. Many haptens enter the cells through passive diffusion and bind to intracellular proteins, which are presented by MHC-I, H-2K^b^, to naive CD8+ T-cells [63]. Presentation to naive T-cells leads to the formation of hapten-specific memory T-cells with the capability to become hapten-specific effector T-cells (CD4+ and CD8+). Thus, these effector cells cause damage and regulate immune responses at the elicitation site [4, 5].

Haptenation also causes the release of endogenous glycolipids that are processed and presented via CD1d to iNKT cells in the liver [65]. In Balb/c and CBA/J mice iNKT-cells become stimulated within 30 minutes via “stimulatory” lipids in the liver and release IL-4 [65–68]. The IL-4, along with haptenated antigen in the circulation [66, 67, 69], stimulates naive B-1 cells within 1 hour to migrate to the draining lymph node and form “CS-initiating B-1 cells,” a distinct class of B-1 cell, that creates hapten-specific IgM [70, 71]. In C57BL/6 mice, however, these iNKT-cells have an inhibitory role [72] as they release IL-4 and IL-13 which, along with T-regulatory cells that release IL-10, suppress the formation and function of the hapten-specific memory T-cells [73, 74]. The differences in function of iNKT-cells are most likely because Balb/c mice have a more Th2-based immune response, whereas C57BL/6 mice have a more Th1-like immune response [72]. Regardless, iNKT-cells play a large stimulatory or regulatory role in CHS.

O'Leary et al. [75] and Paust et al. [76] showed that natural killer (NK) cells induced CHS reactions in RAG^−/−^ mice (devoid of T- and B-cells). Further experimentation [77] showed that liver NK cells are able to transfer CHS to naive animals in 1 hour. Currently, there is no literature on how these NK cells become activated, although one can infer that NK cells are more likely to become activated due to a lack of engagement of inhibitory receptors. Ly49C, found on these hapten-specific NK cells, is specific for H-2K^b^ binding [78]. If the self-protein being presented is haptenated, it may no longer appropriately recognize or bind with the Ly49C, causing NK cells to recognize the cell as foreign. It is likely that DNP-bound MHC will affect Ly49C binding, but this requires experimental verification.

In summary, after hapten application, keratinocytes stimulate dAPC maturation and migration, leading to activation of hapten-specific memory T-cells, iNKT-cells, CS-initiating B-1 cells, and hepatic NK cells. The sensitization phase appropriately primes the immune system to the hapten, so that the elicitation phase can occur quickly and with optimal immune response.

### 2.2. Elicitation Phase of Contact Hypersensitivity

Upon secondary hapten challenge, the elicitation phase of CHS will occur as “early” and “late” events, resulting in swelling and severe damage of the challenged area. The early elicitation phase which peaks within 2 hours of challenge and dissipates by 4 hours seems to be antigen-independent [79], while the late elicitation phase occurs within 24 hours of the challenge and seems to be antigen-dependent [4]. Each of these concepts needs to be considered for understanding hapten-induced tumor-immunity.

#### 2.2.1. Early Elicitation Phase

**Figure 2:**
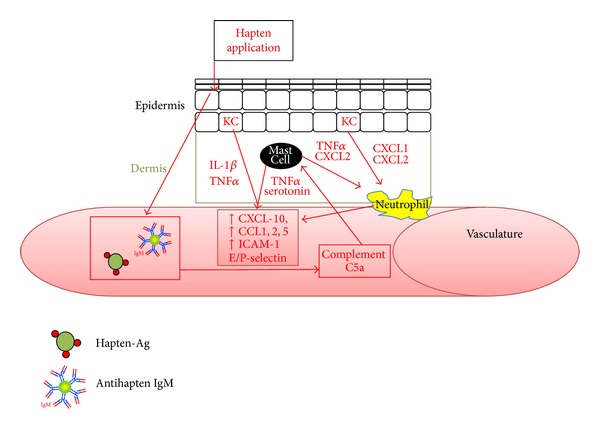
The likely pathway of the “early elicitation” phase of contact hypersensitivity. The red arrows and type indicate the early elicitation phase. Hapten challenge will restimulate iNKT cells to release IL-4, which along with hapten-antigen will stimulate CS-initiating B-1 cells as seen in Figure 1. These cells will release IgM, which will bind to hapten-antigen. This will cause formation of C5a, triggering activation of mast cells to produce TNF*α* and serotonin, increasing immune cell trafficking into the area and TNF*α* and CXCL2 to stimulate neutrophils in the dermis. Neutrophils will also be activated by CXCL1 and CXCL2 released from haptenation of the keratinocytes. Their activation will cause damage at the challenge site as well as more CXCL1 and CXCL2 release, inducing immune cell trafficking to the area as illustrated in Figure 3. Lastly, haptenated keratinocytes will release cytokines to induce immune cell trafficking to the area as depicted in Figure 3.

Figure 2 outlines the steps in the early elicitation phase. Upon hapten-challenge, there is antigen-nonspecific inflammation; iNKT-cells are restimulated by the stimulatory lipids released in the liver, causing them to once again produce IL-4. This release causes the restimulation of CS-initiating B-1 cells to produce IgM against hapten. The hapten-specific IgM and haptenated antigen will go into circulation, form complexes and activate complement C5a [65, 69, 80] through the classical complement pathway. The C5a will then bind to mast cells in the dermis, causing release of serotonin, TNF*α*, and CXCL2. TNF*α* and CXCL2 release will help recruit FasL+, neutrophil + neutrophils to the area. In combination with these neutrophils, TNF*α* and serotonin production by mast cells will cause the release of CXCL-10, CCL1, 2, and 5 from the surrounding tissue and the upregulation of ICAM-1, E- and P-selectin on endothelial cells in the vasculature, leading to hapten-specific T-cell recruitment [4, 61, 62, 81]. Neutrophils are also brought to the area by the release of CXCL1 and 2 from keratinocytes after hapten-challenge and elicit T-cell infiltration [4, 82]. FasL and perforin expression of neutrophils is essential to initiate proper T-cell infiltration, as administration of soluble FasL in the challenge area had similar responses [83]. Keratinocytes are known to release proinflammatory cytokines (IL-1*β* and TNF*α*) upon hapten stimulation [84], causing vascular endothelial cells to upregulate ICAM-1 and P- and E-selectins [4]. In the absence of IL-1 and TNF*α*, CHS is suppressed [85]. Keratinocytes also produce many chemokines that allow for hapten-specific T-cell entry into the challenged area, the most important being CXCL10, which will be bound by the CXCR3 on Th1 cells. The blockade or deficiency of IL-1*β* and TNF*α* reduces CHS by decreasing CXCL10 [4].

#### 2.2.2. Late Elicitation Phase

**Figure 3:**
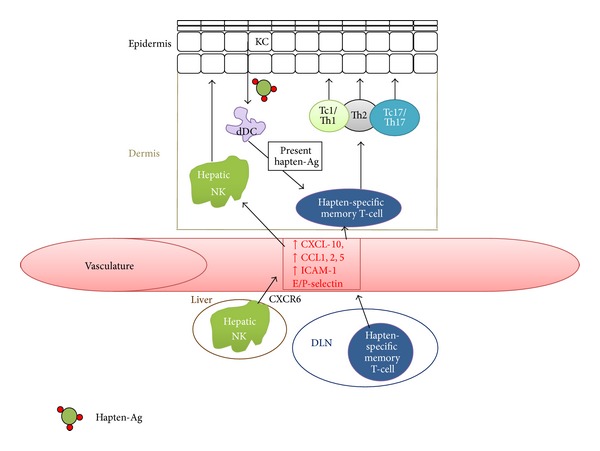
The likely pathway of the “late elicitation” phase of contact hypersensitivity. The red type indicates the “early” elicitation phase and the black arrows indicate the “late” elicitation phase. Hapten-specific memory T-cells will traffic to the hapten challenge site, where they will enter the dermis and divide into multiple different cells subsets. This will be initiated by dermal APCs presenting antigen as well as cytokine release from multiple different cell subsets. The multiple subsets will play different roles in the CHS reaction at the site. Lastly, CXCR6+ hepatic NK cells will traffic to the hapten challenge site and elicit damage.

Figure 3 outlines the steps in the late elicitation phase, which occurs within 24 hours of hapten-challenge. dDCs, LCs, KCs, and endothelial cells process haptenated antigen as previously described and present the antigen to hapten memory T-cells that have migrated to the dermis during the early elicitation phase [86]. Once stimulated in the dermis, memory T-cells will form hapten-specific CD4+ and CD8+ T-cells.

Typically, iNKT cells can either play a stimulatory or inhibitory role that depends on the mouse model used to study iNKT cells, C57BL/6 mice versus CBA/J mice, respectively. In CBA/J mice, iNKT cells can release IFN*γ* that helps to promote CD8+ effector development when working in conjunction with *γδ* T-cells [65, 87]. In C57BL/6 mice, the iNKT-cells release IL-4 and IL-13, which suppress CHS reactions [72], possibly by stimulating a Th2 response. This is in contrast to other strains of mice wherein IL-4 release helps to stimulate CS initiating B-1 cells. *γδ* T-cells seem to “collaborate” with iNKT-cells to elicit CD8+ T-cell-mediated damage during CHS [88]. Upon adoptive transfer with these two cell subtypes, there was a strong ear swelling response at 2 and 24 hours post-DNFB challenge, but if either one was depleted, the ear swelling significantly decreased. This collaboration of iNKT-cells and *γδ* T-cells helps to activate *αβ* TCR+ CS-effector cells [88].

Langerhans cells, once thought to be the main APC of haptenated-Ag, are thought to have more of a regulatory role in the elicitation of CHS. Depletion of epidermal LCs in hapten-sensitized mice elicited greater CHS responses [89] as LCs can suppress CHS responses via CD40-CD40L interactions with CD4+ T-cells causing the release of LC derived IL-10 [90]. Notably, LCs tolerize CD8+ T-cells by activating FoxP3+ T-regulatory cells (T-regs) in mice sensitized with a weak hapten and then challenged with a strong hapten [91]. It is likely that dDCs, endothelial cells, and KCs, not LCs, present antigen to memory T-cells in the dermis during the elicitation phase [5, 92].

Hapten-specific T-cells will traffic to the elicitation site by upregulation of chemokines, selectins, and adhesion molecules and differentiate into their appropriate effector or helper status by a multitude of cytokine signals (from the tissue and activated T-cells) and haptenated-antigen presentation [4, 5, 92]. Honda et al. [4] summarizes the roles of different cytokines in the elicitation phase of CHS and the large difference between the reactions elicited with the haptens trinitrochlorobenzene (TNCB), Ox, DNFB, and fluorescein isothiocyanate (FITC), all which are known to be Th1 haptens except for FITC, which is known to be a Th2 hapten. They further emphasize that the differing effect of cytokines reported in the literature is due to the hapten, animal model, and possibly even the microbiota of the animals in the specific animal facility. We think that haptenation of microbiota will release multiple danger signals, haptenated bacterial proteins, and haptenated bacterial lipid, which can uniquely stimulate different types of CHS reactions through various innate immune responses, iNKT cell responses, and T-cell responses. This concept needs experimental verification.

The “Hapten Atopy Hypothesis,” proposed by McFadden et al. [54], states that haptens delivered a few times by epifocal application will stimulate TLR4 through danger signal release, leading to a Th1 immune response, but repeated and prolonged exposure to haptens will likely shift the response from Th1 to Th2. When TLR4 is stimulated, it will weakly upregulate TLR2 expression to drive Th2 responses, possibly by heat-shock protein ligand upregulation. The repeated exposure of the haptens and weak stimulation of TLR2 will form Th2 cytokines, which will downregulate Th1 cytokines and suppress TLR4 function. This is known as the “danger limitation effect” [54]. Röse et al. [93] indirectly support this hypothesis by showing that different types of hapten challenges, acute (one challenge), subacute (three challenges), and chronic (5–13 challenges) result in different CHS responses. In the chronic exposure versus acute exposure, there is a decrease of Th1 cytokines (TNF*α*, INF*γ*, IL-2, and IL-12), an increase of Th2 cytokines (IL-4, IL-5, and IL-13), and an increase in T-regulatory cytokines (IL-10), indirectly giving support to the “Hapten Atopy Hypothesis”.

There are multiple different T-cell subsets that are involved in the elicitation of CHS-related cellular damage. Classic delayed-type hypersensitivity is CD4+ regulated, and for many years it was assumed that CHS worked the same way. Now it is evident that both CD8+ and CD4+ T-cell subsets are involved in eliciting CHS [94]. The depletion of CD8+ T-cells greatly reduces CHS reactions [95]. Martin et al. [96] showed that CD8+ effector T-cells were the main cells that elicited CHS damage and CD4+ effector T-cells minimally acted as CHS effectors. Along with this notion, hapten-specific CD4+ T-cells are thought to consist of more CD4+ T-regs than effector cells, each having their own effect on CHS responses, inhibitory and stimulatory, respectively [94]. It is likely that both CD4+ and CD8+ effector T-cells work in tandem to elicit damage, as shown in CD4+ and CD8+ T-cell KO mice experiments where both subsets had great impact on CHS responses [97]. It seems that CD8+ T-cells are the main CHS-effector T-cells, and that CD4+ T-cells have a dual role, eliciting minimally the effector function and largely the regulatory function.

CD8+ T-cells elicit damage in the haptenated area during CHS elicitation phase by augmenting cytotoxicity with perforin and Fas/FasL interactions [98]. This interaction seems to induce the apoptosis of KCs [99]. CD8+ T-cells have also been shown to release IFN*γ* and IL-17, which can stimulate neutrophils to draw more CD8+ T-cells to the area by keratinocyte-induced upregulation of chemokines [83, 100]. IL-17 release seems to play an important role in CHS and ACD [101, 102], as Th1/Th17 cells infiltrate ACD areas upon NiSO_4_ application in human patients [103]. These results found in CHS and ACD models show that CD8+ T-cells and possibly Th17 cells are crucial players in CHS reactions.

T-regulatory cells down-regulate contact hypersensitivity by using the IL-2 produced from hapten-specific CD8+ effector cells [104]. CHS-associated T-regs traffic to the inflamed site during the elicitation phase [74] and likely inhibit CHS by CTLA-4 and CD86 interactions between T-regs and CD8+ T-cells, as treatment with anti-CTLA-4 antibody increased CHS responses [105]. They also inhibit CHS by IL-10 release, which is known to suppress CHS [106] and block entry of hapten-specific effector T-cells into the challenge site [73]. Taken together, T-regs play a large role in CHS regulation and are important when considering hapten-induced tumor regression.

Extensive studies were performed by Hans Ulrich Weltzien's group from 1992 to 1997 looking at the TCR specificities of CD4+ and CD8+ T-cells and the way in which haptenated protein is presented to T-cell receptors (TCRs). They showed that trinitrobenzene sulfonic acid (TNBS)-like haptens are H-2K^b^ restricted [64]; haptenated Ag can be processed intracellular in the ER/Golgi to be presented by MHC I [63], and trinitrophenyl (TNP)-specific T-cell clones were able to recognize haptenated and unhaptenated portions of designed tryptic fragments of TNP-octapeptides [107]. TNP-specific CD4+ T-cell clones were able to recognize many different TNP-modified peptides, as long as TNP was present [108]. These papers suggest the ability of hapten-specific CD8+ clones to recognize unhaptenated portions of amino acid chains, whereas hapten-specific CD4+ T-cells only recognize haptenated protein.

*γδ* T-cells and iNKT-cells were shown to work together to release IFN*γ*, which would stimulate a Tc1/Th1- like response [88]; however, they were shown to inhibit CHS reactions during elicitation by hindering the development of hapten-specific CD8+ T-cells [109]. *γδ* T-cells played a role in eliciting dinitrochlorobenzene (DNCB)-induced CHS in lambs [110]. Recent unpublished work by Xiaodong Jiang et al., presented at “The American Association of Immunologists Conferences in May of 2013,” focuses on the dermal *γδ* T-cells in terms of how their depletion suppresses CHS reactions. It seems that IL-17 dermal *γδ* T-cells are important in inducing CHS reactions. The involvement of dermal *γδ* T-cells during elicitation is unclear and needs further study.

Recent studies have unraveled the ability of NK cells to induce CHS reactions. First described by O'Leary et al. [75] and Paust et al. [76], CHS was induced in a RAG^−/−^ mouse (lacking B- and T-cells) with the assumption being that no ear swelling would be seen; these animals got an ear swelling reaction close to normal. The responsible cells were NK cells as seen by IL-2R^−/−^ mice and antibody depletions. Using adoptive transfer systems, it was seen that these NK cells were hepatic, expressed Thy-1, Ly49c, and CXCR6 and could elicit CHS responses 4 months after sensitization. L-, P-, and E-selectins and NKG2D were found to play an important role in NK-mediated CHS reactions [75, 76]. These observations were furthered by Carbone et al., [111] who looked at a distinct CD3−, CD16−, perforin+, CD56^high^, CD16−, and CD62L− (noncirculating) NK cell populations that produced IFN*γ* and TNF*α* in Nickel-challenged ACD regions of humans. Unexpectedly, these NK cells did not elicit a memory-like response as previously described but did contribute to keratinocyte apoptosis; this could be a mouse versus human phenomena [111]. Majewska-Szczepanik et al. [77] confirmed the presence of NK cell-mediated CHS in mice devoid of B- and T-cells, although the response was markedly diminished compared to wild-type (WT) mice. These cells produced IFN*α*, IFN*γ*, and IL-12, were Thy1+ and MAC1+ (fully licensed), CXCR6-dependent, and could elicit a CHS reaction in as little as 1 hour after transfer from a sensitized to naïve animal [77]. Likely uncertain of this body of results, Rouzaire et al. [112] did a comparison of T-cell-mediated to the NK cell-mediated reactions using the “classical” CHS protocol with DNFB; they showed that the NK cells failed to create a genuine CHS response in RAG2^−/−^ mice, as the DNFB ear challenge did not require sensitization to elicit an ear swelling response. They confirmed O'Leary et al.'s [75] observations by performing similar adoptive transfer experiments of NK cells and showed that the responses were similar to transferred CD8+ T-cells. However, the recall response of these transferred NK cells upon a second hapten challenge was much weaker and short-lived than that of transferred CD8+ T-cells and there was little CD45.1+ T-cell infiltration into the challenged site in the NK cell-transferred mice [112]. It seems as though NK cells play some sort of role in CHS, although they may only be able to elicit true CHS reactions in adoptive transfer settings and may only help to elicit damage at the haptenation site.

## 3. Drug-Induced Autoimmunity versus Hapten-Induced Autoimmunity

There are many common allergens that cause CHS: metals likes Nickel or Gold, certain antibiotics like Neomycin, topical anesthetics, natural compounds such as Urushiol, the irritant in poison ivy, and many more. These all act directly as haptens, inducing a CHS-like reaction in the skin. There are instances where metabolizing a drug or chemical can lead to autoimmune-like responses, idiosyncratic drug reactions. This is when a drug's metabolite acts as a hapten and binds to cellular proteins, eliciting an immune response and antibody production to the metabolite-protein conjugate, the metabolite alone, and the protein alone [129]. These drugs are prohaptens, or chemicals that are not protein-reactive unless metabolically activated to the electrophilic state [1]. A common example of this is Penicillin-induced hemolytic anemia [7]. When the penicillin enters the body, it is metabolized in the liver and forms Penicillenic acid, similar to the hapten Oxazolone, which then covalently binds to red blood cells (RBCs) [7]. Antibodies (IgG) can form against the hapten-coated RBCs, which are then killed by antibody-dependent cellular cytotoxicity (ADCC) and cleared by macrophage opsonization. Hydralazine, a hypertension drug, is known to cause drug-induced lupus (DIL) through its metabolites. It was seen that hydrogen peroxide and other oxidants from the lungs react with hydralazine to produce metabolites that bind to self-protein. About 5% of the patients who take this drug develop DIL-like symptoms [130, 131]. There are several other examples of drug-induced autoimmunity in several different contexts, most involving the binding of a drug or its metabolite to self-protein inducing antibody production. In all cases, the drug or metabolite acts as a hapten to induce autoimmunity.

The autoimmune inducing capabilities of haptens have been shown experimentally. Paul et al. [2] showed proof of principle experiments that haptens could allow the immune system to overcome peripheral tolerance. By injection of haptenated-BSA, BSA-tolerized rabbits were able to induce the production of antibody towards the hapten, the BSA, and the conjugate. Haptens have been shown to induce hapten-specific CD8+ T-cell cross-recognition of haptenated and unhaptenated octapeptides as previously described [107]. Kang et al. [132] showed hapten-mediated autoimmunity experimentally in hen egg lysozyme (HEL)-transgenic (Tg) C57BL/6 (B6) mice that were immunized with HEL or hapten-modified (phosphorylcholine [PC]) HEL (PC-HEL). Hen egg lysozyme immunization failed to induce antibody responses against HEL in the transgenic animals, but the PC-HEL generated large amounts of anti-HEL antibody. This break in tolerance was by T-cells seen through T- and B-cell depletion and adoptive-transfer experiments. This concept is similar to that seen in CHS. Lastly, PC-HEL was better at generating HEL epitopes for T-cell recognition following antigen processing. They suggest that the “generation of new immunogenic epitopes of self-antigens may result in breaking self-tolerance and lead to the production of autoantibodies” [132]. Despite these examples, none of these papers showed the ability of these reactions to induce immune damage, as this would be indicative of autoimmune disease. Experimentally induced autoimmunity seems to be a hapten-dependent reaction that does not occur in the absence of the hapten.

Clearly, the main use of haptens is to study CHS. The unique property of haptens to induce immune reactions against self-peptide has been utilized in many other settings besides CHS. Haptens have been commonly used to induce acute and chronic IBD in rats and mice using the haptens 2,4,6-trinitrobenzene sulfonic acid or 2,4-dinitriobenzene sulfonic acid (DNBS) to induce immune reactions in the intestine [11–15]. te Velde et al. [14] reviewed the models of TNBS-induced IBD, clearly stating many of the problems present in the field. IBD reactions seem to be hapten-dependent, and the hapten does not induce autoimmune reactions to the intestine once it is out of the animals' system. Haptens have been used to treat drug addiction. Ennifar et al. applied for a patent [9] for a novel hapten-carrier conjugate that stimulates the production of antibodies against nicotine. These antibodies could be used to treat nicotine-addicted patients, as they passively lower the nicotine levels in the serum and brain. A similar idea was tried using a novel hapten-conjugate, 6-glutarylmorphine-Keyhole Limpet Hemocyanin (KLH), conjugate that induced antibodies against morphine and heroin in rats. The treatment increased rat movement and attenuated other drug-induced behaviors, compared to the control group, in morphine and heroin addicted rats; this was associated with antibodies against the morphine and heroin. This treatment likely induced tolerance to the drugs [10]. These methods have not been extensively studied, making long-term dependence on the haptens unclear.

## 4. Applying Haptens and Contact Hypersensitivity to Antitumor Immunity

Clearly, haptens have been used in many contexts to study certain diseases and induce responses against certain malignancies. The properties of haptens to induce reactions are fascinating, although it seems as though these reactions may be hapten-dependent, and many will wane as the hapten is cleared. Despite this, the ability of haptens to induce reactions against self-protein, even if haptenated, is a unique property that make haptens tantalizing targets for cancer immunotherapy. In the following sections, we will review how haptens have been used to treat tumors, their advantages and disadvantages, the challenges present in the field, and possible directions of study to further the field.

### 4.1. The Four Concepts of Hapten-Mediated Antitumor Immunity

The use of haptens to induce tumor regression is not a new one, as many groups have attempted several different methods of hapten-mediated tumor regression. There are four overarching concepts involving the use of haptens to induce tumor immunity. (1) The tumor is removed, haptenated* ex vivo*, and injected back into sensitized animals or patients [21–31]. (2) The tumor is haptenated* in situ* (in the tumor) [32, 33]. (3) The tumor area is haptenated epifocally (at the tumor site) to induce a CHS-like reaction [34–42]. To note, this method has only been utilized for cutaneous skin cancers that can invade the epidermis or dermis, as CHS reactions require these. (4) ADCC reactions at the tumor site can be induced by intraperitoneal (i.p.) or subcutaneous (s.c.) administration of antigen-hapten conjugates in mice and patients, respectively with antigen-receptor high tumors [43–47]. These concepts (Table 1), the problems and holes present, and our interpretation of the possible antitumor mechanisms occurring are reviewed below.

### 4.2. *Ex Vivo* Haptenation to Mediate Tumor Regression

Many groups have utilized* ex vivo* haptenation to induce tumor regression in mice and humans. Hamaoka et al. [21] were the first group to use* ex vivo* haptenation as a cancer immunotherapy in mice. They used X5563 cells, a plasmacytoma cell line syngeneic to C3H/HeN mice previously shown to generate “killer” T-cell activity without inducing helper T-cell activity against tumor-associated transplantation antigen (TATA) and still grow. They immunized mice with hapten-modified X5563 cells to amplify helper T-cell activity, and augment killer T-cell responses to the TATA. They primed mice intraperitoneal (i.p.) with trinitrophenyl (TNP)-bound mouse gamma globulin (MGG) in order to generate TNP-specific T-cells. Six weeks later, they immunized mice i.p. with TNP-bound X5563 tumor cells, using TNBS, generating killer T-cells against X5563 and TNP-X5563 tumor cells; this did not occur in mice primed with unhaptenated tumors. They further amplified this response with a pretreatment of TNP-D-GL to ablate TNP-suppressor cell activity. Mice were given the full treatment (TNP-D-GL pretreatment, three days after TNP-MGG immunization, six weeks after immunized i.p. with TNP-X5563 cells once a week for five weeks) and then given a lethal dose of the X5563 cells. The tumor growth was greatly decreased and the mean survival time of the mice increased by 10 days posttreatment. This study only examined the tumor growth for 15 days, so it is likely that the tumor was able to proliferate and grow at further time points. This system is a nice proof of principle but has very little clinical application because it is a lengthy prophylactic treatment that minimally delays tumor growth and the effect of this treatment on an established tumor was not studied. Regardless of this, they showed that modification of TATA with hapten-induced TNP-reactive helper T-cells, which could amplify killer T-cell generation, resulting in slowed tumor growth and an antitumor immune response* in vivo*.

Fujiwara et al. [22] took Hamaoka's model and applied it to a BALB/c-LSTRA leukemia tumor system. They primed mice with TNP-D-GL, three days later, immunized mice with TNP-MGG, and six weeks later, i.p. injected TNP-LSTRA cells three times in two-week intervals. Syngeneic T-cells were stimulated* in vitro* by co-culturing them with TNP-LSTRA cells for five days. These cells showed significant lysis of LSTRA cells* in vitro*. The TNP-primed splenocytes were collected, mixed with viable LSTRA cells to perform* in vivo* tumor neutralization assays by intra-dermally (i.d.) injecting the mixture into TNP-sensitized Balb/c mice. This stopped tumor formation for at least 11 days after inoculation. Despite not showing the effect of this treatment on tumor cell challenges or established tumors, this study highlights the proof of a principle that anti-tumor immune responses can be generated with* ex vivo* haptenation of tumor cells.

Flood et al. [23] investigated* ex vivo* TNP-modification, using TNBS, of regressor and progressor tumors to cause tumor rejection of unmodified progressive tumor cell lines in mice. They created a system of tumor inoculation rejection in C3H/HeN mice using primary s.c. immunization of TNP-bound 1591 regressor fibrosarcomas, followed 28 days later by a secondary immunization of a TNP-bound 3152 progresser fibrosarcoma and tertiary challenge of unmodified-3152 progressor cells. This resulted in slowed growth of 3152 progressor tumors for up to 30 days. The resistance to progressor tumor cells was adoptively transferred with total splenocytes to naïve animals. By antibody depletion, it was seen that Lyt-1-2+ T-cells and Lyt-1+2- T-cells expressing nonclassical helper T-cell phenotypes elicited the resistance. Thus, they established that haptenation could enhance immunity towards “weak” tumor-associated antigens by TNP-modification, despite the eventual progressor tumor growth. It would be interesting to see what would have happened if they had used a cytotoxic hapten, like TNCB for their immunizations, as hapten-mediated cell death may have elicited better immune response, or if they had sensitized the animals to TNP before vaccination, as this may have enhanced the immune response to the haptenated cells.

Berd et al. [24, 26, 28, 30] utilized the* ex vivo* haptenation as well as* in situ* haptenation mouse studies by Fujiwara et al. [32, 33] as the basis for clinical trials using* ex vivo* tumor cell haptenation as a primary treatment for metastatic melanoma or as an adjuvant treatment after surgical resection of nodal metastases in stages III and IV metastatic melanoma patients. Two weeks before vaccination, patients were pretreated with cyclophosphamide (CY) and 2 days later sensitized to 1% DNFB. Patients were treated with CY three days before the DNP vaccination; this was repeated every 28 days. Cyclophosphamide has long been known to enhance CHS-like responses as it decreases the percentage and number of CD4+ CD25+ T-regs that suppress the induction of CHS [133]. The DNP-vaccine was made by surgical resection of primary melanoma, irradiation, modification with DNFB, and intradermal injection back into patients along with Bacillus Calmette-Guerin (BCG), a known cancer immune adjuvant [134]. Forty-six patients with measurable metastases were treated, resulting in 20 patients with clinically evident inflammatory responses in nodal, subcutaneous, or intradermal tumors. These tumors had increased CD8+ T-cell infiltration, compared to prevaccination tumors, which strongly expressed HLA-DR and CD69 suggesting activation. In addition, 140 T-cells clones were created, 70 of which could kill autologous melanoma cells* in vitro*. It is commonly seen that tumor-infiltrating lymphocytes (TILs) are able to kill tumor cells* in vitro* once stimulated [135], so it is unclear if the DNP-vaccine was responsible for this cytotoxicity. Of the 40 evaluable patients, only five had clinical responses, four complete and one partial, with a median duration of 10 months. One patient remained melanoma free for 10 years after treatment. In the same publication [30], Berd et al. looked at the antitumor effects of DNP vaccination as a postoperative adjuvant therapy; they compared 41 patients treated with the vaccine after surgical resection to 22 patients who received surgical resection with administration of unhaptenated cells. They used the nodal melanoma metastases to prepare the vaccine. Patients received i.d. DNP vaccinations in 4-week intervals and CY was given 3 days before the first 2 vaccinations. The results correlated to a 3-year disease-free survival of 59% for the patients vaccinated with hapten-melanoma cells compared to about 24% for the patients that received the unhaptenated melanoma cells, suggesting that a good clinical response depended on the haptenation of the injected melanoma cells. Neither the immune-correlates nor tumor inflammation for this trial were fully corroborated. This was only a short and small study, so it is hard to make concrete conclusions from this, although it indicates that DNP-vaccination is more useful as a postadjuvant therapy with less tumor burden. Of note, the control unhaptenated vaccine used in this study was not included for any of the subsequent trials [24–29].

Sato et al. [29] studied the immune response induced by the DNP-modified vaccine in these trials. They collected serum and peripheral blood lymphocytes (PBL) from 27 patients before DNFB sensitization (day 0), after DNFB sensitization (2 weeks), after two vaccinations (day 63), after four vaccinations (day 119), after six vaccinations (day 175), and after eight vaccinations (day 231) for immunologic study. There were DTH responses to DNP-modified autologous PBL and melanoma cells, although DTH responses to unmodified cells were not reported. They detected the development of anti-DNP antibody in 24 of 27 patients that was not induced by DNFB sensitization alone. Peripheral blood lymphocytes from 8 of 11 patients were stimulated with “DNP-modified autologous lymphocytes”* in vitro*; there was no response to unconjugated or TNP-conjugated autologous lymphocytes. CD8+ and CD4+ T-cells from these stimulated PBL were able to respond to DNP-modified lymphocytes, however, only CD8+ T-cells could respond to DNP-modified melanoma cells. None of these cells were able to respond to unmodified autologous PBL or TNP modified-autologous melanoma cells. These responding CD8+ T-cells produced high amounts of IFN*γ* and could kill DNBS-modified autologous melanoma cells; cytolytic activity to unmodified cells was not examined. In their discussion, the authors mention that they did not see an* in vitro* reaction to unmodified melanoma cells, but state that their clinical findings still hold true and that there is inflammation of distant tumor sites. They propose that in humans, the majority of T-cells are going to be reactive to DNP-melanoma, but there may be a small subset of cells that are able to react with the unmodified melanoma cells. Of note, this has yet to be demonstrated. In this regard, they showed no reaction of the responder T-cells to unmodified melanoma cells and did not study how these responder cells would specifically respond to modified or unmodified melanoma antigens (i.e., gp100 or HMW-MAA) that are known to elicit a T-cell response [29, 136].

Sato et al. [27] further observed that the DNP-specific T-cells from patients were able to respond to small DNP-modified peptides associated with the MHC, although these responses were limited to one HPLC peptide fraction of autologous melanoma. Of note, these T-cells did not respond to unmodified peptide fractions. This paper suggests that these T-cells are not going to respond to unmodified melanoma cells, which suggests that the hapten-specific T-cells are not affecting the tumor cells and may not be the only factor in the inflammation of distant metastases as concluded by Berd et al. [24, 26, 28, 30].

In 1997, Berd et al. [28] used the DNP-vaccine as a postsurgical adjuvant treatment after resection of nodal melanoma metastases in 62 patients. They observed 45% relapse-free survival in stage III melanoma patients compared to historical controls, stage III patients from an ECOG IFN*γ*+ resection study and an ECOG resection only study, which showed 34% and 22%, respectively. The HLA class I phenotype (A3+A2−), number of metastases (lower), age (>50 years old), DTH to unmodified autologous melanoma, and tumor inflammation seen in patients posttreatment were all positively correlated to overall survival. There were no experiments or discussion of the antitumor mechanism occurring in the patients except for histology of resected tumors posttreatment showing lymphocyte infiltration. The data is difficult to interpret as the controls groups were historical controls, albeit the fact that the inclusion of patients in the trial was based on poor prognosis and no patient was excluded that had extranodal extension of melanoma. However, the results would have been clearer if there had been a control group that only received unhaptenated tumor cells, as done in their earlier trials [30], to ascertain the importance of the haptenation in eliciting a response. Further immunogenic studies are necessary as well as studies with appropriate controls to unravel the efficacy of haptenation. In 2004, Berd et al. [24] extended the 1997 study to 214 patients with 5-year overall survival of 44%. Patients with DTH responses to unmodified autologous melanoma had a 5-year overall survival of 59%, double that of the DTH-negative group, whereas DTH to DNP-modified melanoma gave no overall survival benefit. They retrospectively observed that a baseline skin test with the DNP-vaccine before the start of treatment (on day −8 and −3) acted as an induction dose, which increased the overall survival of patients. As much of the data was based on clinical observations, there was no direct immune correlation between the vaccine and the tumor responses [24, 28].

Berd et al. [26] used the DNP-vaccine to treat pulmonary melanoma metastases in 97 stage IV patients. In this study, 11 responses out of 83 evaluable patients, two complete, four partial, and five mixed, were observed. The study describes several case reports of patients who had tumor regression of pulmonary metastases. Along with this, only 27 of 83 (33%) patients had tumor inflammation following the DNP-vaccine; lymphocytes and CD3+ cells infiltrated these tumors. Beside this, there were no immune correlates studied in this paper and it is difficult to know whether treatment caused the observed clinical outcome.

Manne et al. [25] studied the TCR rearrangement of the associated TILs in inflamed melanoma metastases after the DNP-vaccine. They observed that 9 of 10 inflamed tumor samples had dominant peaks in the same V*β* families. However, it was not tested if these TCRs were melanoma antigen-specific or if they could recognize unmodified melanoma cells.

The clinical trials using DNP-vaccine [24, 26, 28, 30] lack immunologic data linking the DNP-vaccine to an immunologic response at unmodified melanoma sites. The main focus of these papers seems to be T-cell responses, when it is now clear that multiple different cell subsets are involved in hapten responses; NK cells, iNKT-cells, Mast cells, B-1 cells, and neutrophils should have been considered in this study and could have been causing the distant tumor inflammation they observed. Along with this, there was no direct comparison of the DNP-treatment versus same the vaccine without DNP-modification after the first clinical trial, making it hard to know the efficacy of the subsequent trials. Lastly, there is no data showing the efficacy of the* in vitro* haptenation, as it is likely that there were a small percentage of unmodified cells present in the vaccine that could have elicited the inflammation seen in the tumors.

Sojka et al. [31] extended these clinical trial protocols as a postsurgical adjuvant therapy for 410.1 mammary carcinoma-bearing Balb/c mice. Tumors were surgically excised before vaccination. Four to six days after excision, CY was i.p. injected followed by an s.c. injection (every 10 days for the duration of the experiment) of either unmodified or DNP-modified, irradiated 410.4 tumor cells with BCG. Importantly, the clinical trials by Berd's group injected the vaccine intradermally [24–30], whereas Sojka et al. [31] injected subcutaneously, which greatly alters the immune responses occurring. The DNP-modified treatment resulted in about 40% relapse-free survival of the mice, while the unmodified treatment was about 20%. They looked at multiple different parameters of the DNP vaccine to see what portions of the treatment were important and to study some immune correlates to the vaccine. There was a significant increase in relapse-free survival when using CY pretreatment. Relapse-free survival decreased with the depletion of CD4+ or CD8+ T-cells. The draining lymph node cells from mice showed a significant increase of IFN*γ* production when given DNP-modified versus unmodified vaccine. Lastly, there was a significant decrease in relapse-free survival when neutralizing IFN*γ* or TNF*α*. Surprisingly, the mice in this study were not sensitized to DNP before immunization, as done in Berd et al.'s clinical trials [24, 26, 28, 30] and shown to be crucial for antitumor responses. This study demonstrates a clear immunologic correlation between the hapten-modified vaccine and relapse-free survival of mice with mammary cancer, but does not fully explain the mechanism of this antitumor immune response. Importantly, this model is not representative of the clinical trials as it uses a different injection method than the clinical trials and does not use DNP-sensitization, likely eliciting a different response.

### 4.3. Plausible Immunologic Reactions Linked to *Ex Vivo* Haptenation

The immune responses occurring in* ex vivo* haptenation that elicit antitumor immunity are dependent on the injection site. Miller and Claman [142] and Mekori and Claman [143] showed that i.v. injection of DNP-modified cells induced tolerance to CHS-like reactions in mice. They further observed that the repeated i.v. injection of haptenated cells induced “desensitization” [143, 144]. Considering this issue, the anti-tumor immune studies dealt with administration of* ex vivo* haptenated-cells in three ways, i.p. (Hamaoka et al. [21] and Fujiwara et al. [22]), i.d., (Berd et al. [24, 26, 28, 30], Sato et al. [27, 29], and Manne et al. [25]), or s.c. (Flood et al. [23] and Sojka et al. [31]) injection, most likely to avoid tolerance and to take advantage of different immune responses. However, much of the mechanisms described below are not supported by experimentation, only by inference.

The mechanism of antitumor immunity after* ex vivo* haptenation by i.p. injection is probably similar to the classic hapten-protein response. B-cells in area of injection likely recognized the hapten-protein conjugates. Sensitization with the TNP-MGG conjugate causes initial recognition by B cells. The conjugate would have been taken up and processed, upon which the conjugate-protein would be presented to CD4+ helper T-cells causing cross-activation of both the T-cell and the B-cell. This would have caused the B-cell to produce antibodies against the hapten, the protein, and the conjugate [2] and would have caused the CD4+ T-cell to elicit hapten-antigen specific responses [108]. It is also possible that the antihapten/antitumor IgM and IgG bound to haptenated cells, inducing ADCC and/or opsonization by macrophages. In terms of the work by Hamaoka et al. [21] and Fujiwara et al. [22], the sensitization would form B-cells specific for the TNP, MGG, and TNP-MGG. Upon secondary stimulation with TNP-X5563, the TNP-specific B-cells would quickly recognize the TNP and produce hapten-specific IgM, binding TNP-X5563 cells and allowing for opsonization by macrophages or ADCC. This would have produced TNP-modified X5563 tumor antigens that could have been recognized and processed by the hapten-specific B-cells causing further cross-activation and the formation of CD4+ T-cells specific for X5563 cells. These CD4+ T-cells would have likely produced Th1 cytokines, like IFN*γ* and IL-2, stimulating X5563-specific effector T-cell clones already present in the animal allowing for cytotoxic responses to the tumor. It is also distinctly possible that one of the reasons their treatment was not very effective was because they modified the tumor cells with TNBS, which keeps cells viable [145]. This means that hapten-modified or unmodified protein was not immediately available for B-cells to process and elicit a quick reaction. Using a toxic hapten, like TNCB [146], may have made antigen more readily available for B-cells to process due to the tumor cell death.

The antitumor mechanism that was elicited from s.c. administration of* ex vivo* haptenated cells is more difficult to interpret as these studies used very different mouse models and delivery systems. Flood et al.'s [23] method likely induced a response similar to that described with the i.p. injections. When injected into the animal, the regressor tumor cells likely had cytotoxic T-cells that were specific for them and could clear the tumor cells when injected into the animal. If the regressor tumors were TNP-modified, it would have allowed for the release of TNP-bound proteins from these regressor cells that were being actively killed. This would have helped B-cell and CD4+ T-cell cross-activation as described with i.p. injections. Upon second immunization, hapten-specific B cells would have recognized the TNP-bound progressor cells and caused cross-activation with CD4+ T-cells, creating B-cells and CD4+ T-cells against the progressor tumor. The activation of tumor specific B-cells would have caused antibody formation against the tumor cells, potentially inducing ADCC or opsonization. The CD4+ T-cells would have provided costimulation to cytotoxic T-cells, which are otherwise unable to clear the progressor tumor. These in combination would have likely created the tumor resistance seen in Flood et al.'s [23] study. As stated above, using a toxic hapten may have made the antigen more readily available for B-cells to process due to the tumor cell death.

Sojka et al.'s [31] method of s.c. injection is much different, as it acts as an adjuvant therapy for any established metastases after surgical resection of the primary tumor. Importantly, the removal of the tumor could have been the priming step to the immune system as surgical resection of a primary tumor can reverse tumor-induced immunosuppression, even in the presence of metastases [147]. Their vaccination protocol killed the cells via irradiation and DNFB modification [146, 148], so it is likely that there would have been much DNP-modified protein available. The vaccine was also mixed with BCG, which stimulates the innate immune system. The actual vaccination protocol probably would have induced a similar response as Flood et al.'s [23] once the treatments were started. They delivered hapten-modified protein to the immune system, which would have stimulated a strong immune response due to repeated vaccination, hence the enhanced survival of mice with established tumor metastases. The sensitization occurred from DNP-modified tumor cell protein from the first injection, inducing cross activation of B- and CD4+ T-cells as described above and subsequent responses against the tumor [31].

The protocol of i.d. injection of hapten-modified tumor cells by Berd et al. [24, 26, 28, 30] appears to be the most appropriate* ex vivo* haptenated-vaccine administration as CHS-like immune responses will likely occur. In the clinical trials, patients were mostly sensitized before administration, allowing for the vaccination to induce CHS elicitation-like reactions (Table 2 and Figure 2). Importantly, these reactions will not be as strong as typical CHS reactions due to the lack of skin haptenation and subsequent innate immune responses, as the haptenated cells were intradermally injected. The danger signal release from skin haptenation would not have occurred; meaning restimulation of keratinocytes and dermal APCs would have occurred more slowly, causing less cytokine release. Also, no “early” elicitation of CHS-initiated mechanisms would have occurred, as iNKT-cells specific for haptens would not have become activated, implying that hapten-IgM from CS initiating-B-1 cells would not be produced. Decreased keratinocyte and CS-initiating B-1 activation would reduce stimulation of mast cells and neutrophils, lowering chemokine, selectin, and adhesion molecule upregulation in the vasculature and the trafficking of hapten-specific T-cells and NK cells to the area. Despite this, there would have been involvement of hapten-specific T-cells and hepatic NK cells, as the BCG will cause stimulation of the innate immune system allowing for recognition of haptenated-antigen. BCG likely initiated peripheral immune responses unrelated to the hapten vaccine, which might have slightly inhibited the response, as the immune system could have been “busy” mounting a new response. It may have served Berd et al. [24, 26, 28, 30] to epifocally apply DNFB to the site of the i.d. injection, eliciting a CHS reaction that could have exposed the vaccine to the immune system in a CHS context. Despite all this conjecture, it is hard to know how an antitumor response would have formed as i.d. injection would elicit a hapten-specific immune response and the DNP-vaccine trials did not show much experimental evidence of antitumor immune responses occurring from the vaccination.

Another important concept to consider is that haptenation in this fashion may not have induced a bystander effect (kill distant, unmodified tumor cells via immune responses) since the process seems to be hapten-dependent. Much of the justification for the work done was based on Weltzien's group's papers between 1992 and 1997, as earlier described [63, 64, 107, 108]. In this work, they saw the ability of hapten-specific CD8+ T-cell clones to recognize and respond to hapten bound and unbound portions of small tryptic fragments of proteins suggesting some cross-reactivity of the cells. An overarching assumption is that this will be true for naturally processed proteins, like that present in the clinical trial treatments using* ex vivo* haptenation. Sato et al. [27, 29] show that DNP-specific TILs from DNP-vaccinated patients (that were not present before vaccination) were specific for only two peptide fragments from a melanoma peptide library and these fragments had to be DNP-modified. To note, no stimulation occurred with unmodified cells. Despite clinical observations of bystander effects, it is very hard to decipher what is occurring since there is not much experimental evidence in support of this claim. As stated before, it is possible that unmodified melanoma cells injected into patients with this vaccine induced an immune response along with the DNP-protein response, leading to tumor inflammation and some antimelanoma immune response. Despite all the work done on* ex vivo* haptenation, as alluded above, there is little experimental evidence to suggest that the vaccination induces direct antitumor effects even though the DNP-vaccine trials show survival impacts in patients. Along with that, the treatment is expensive and very time consuming and relies on the removal of a tumor mass, making it an untenable option.

### 4.4. *In Situ* Haptenation to Mediate Tumor Regression

Fujiwara et al. [32] seemingly abandoned their* ex vivo* tumor cell haptenation immunization for* in situ* haptenation of established tumors. They created a tumor regression model in C3H/HeN X5563 plasmacytoma tumor-bearing mice (dermal) by intratumoral injection of TNCB in TNCB sensitized C3H/HeN mice. As before, they suggested that haptenation would augment TATA helper T-cell responses to generate more powerful killer T-cell responses. They established the following method of tumor regression; pretreatment of CY, 2 days later TNCB sensitization, 5 weeks later implantation of tumor cells, ~6 day after implantation intratumoral injection of 0.15 mL 0.5% TNCB into tumor masses between 7 and 10 mm in diameter. Importantly, splenocytes from sensitized mice caused* in vitro* lysis of TNP-X5563 cells, while unprimed mice splenocytes did not. TNCB ear challenge after 5 weeks induced a response, suggesting appropriate sensitization. The spleen cells from tumor-bearing mice, stimulated* in vitro* with irradiated TNP-X5563 tumor cells, along with the addition of TNP-helper cells, resulted in appreciable augmentation of anti-X5563 cytotoxic T lymphocyte (CTL) responses. Of the fully treated mice, >50% of them had complete tumor regression, as measured by the absence of myeloma protein from the blood serum 45 days after treatment. Of these animals, 90% of them rejected a secondary tumor challenge of 1/10th the original tumor cells, although the data is not shown. An issue of this study is that 0.15 mL of solution was injected into tumors regardless of their size, meaning that smaller tumors would have increased haptenation and* vice versa*. It is possible that the animals that responded all had smaller tumors, although this was not recorded or mentioned in the study. Large injection volumes could potentially cause the tumor microenvironment to be destroyed, causing tumor cell spillage into the animal. The destruction of tumors sites could have also induced enhanced DNP-tumor reactions by the animal due to better availability of the tumor cells. Although this was the first model of* in situ* haptenation of a tumor and subsequent tumor regression, the mechanism remains unclear.

Fujiwara et al. [33] furthered their method by showing secondary challenge and neutralization data as well as repeating it in multiple model tumor systems. They repeated their results in the X5563 system, showing that 4 of 5 mice had tumor regression. Myeloma protein was not present in their serum for up to 2 months after regression. They challenged mice with only 10^5^ X5563 cells (1/10 of the primary inoculation) intradermally showing that 11 of 12 of the mice could resist the tumor, compared to 0 of 10 in naïve mice or 2 of 10 in surgically resected mice (this data was not shown in their previous paper). Conversely, they do not show the tumor growth in these injections and use the word “resistance,” which would imply that the tumors still grew after the challenge, even if the treatment slowed their growth. This is supported by Winn assays at low E : T ratios that shows slight tumor growth 12 days after secondary tumor challenge. In addition, Fujiwara et al. [32] established TNP-mediated tumor regression in mice with methylcholanthrene (MCA)-induced transplantable tumor cells (MCH-1-A1) and MCA-induced autochthonous tumors using similar methods. The MCH-1-A1 showed similar primary tumor regression as that of the X5563 model. For the inducible system, 11 of 25 of the animals had tumor regression for up to 12 weeks. To note, many of the regressed tumors stayed at a constant size or slowly decreased in size for about 5 weeks after TNCB injection, there after dramatically increasing or decreasing in size. The reproducibility of tumor regression in different tumor models is encouraging, but the fact that the secondary tumor challenges were only resisted and not rejected suggests that this method may not induce strong antitumor immune responses and may be hapten-dependent [33].

### 4.5. Plausible Immunologic Reactions Linked to *In Situ* Haptenation

*In situ* haptenation offers the most challenging explanation of what occurs, as it relies on the immune cells present inside the tumor microenvironment to elicit responses. It is likely that the haptenation of tumor cells will cause massive amounts of cell death, as typically seen from haptenation [146], of not only the tumor cells but any of the stromal cells associated with the tumor. This will cause the release of many danger signals and haptenated protein, which will stimulate APC present in or near the tumor, tumor-infiltrating dendritic cells. These dendritic cells may migrate to the dLN where it is possible that it will stimulate a T-cell response to the tumor antigen [149]. Fujiwara et al. [33] concluded that two mechanisms might have occurred to cause tumor regression: (1) a DTH response to the TNP-modification of tumor cells, eliciting anti-TNP CTL, B cells, and DTH responses in the tumor site or (2) the bystander effect of anti-TNP CTL by amplification of anti-TNP helper T-cell activity. Neither of these mechanisms has been confirmed, but the extensive mechanisms of CHS were not as clear in 1984, so it is likely that the mechanisms are far more complicated than that, and that there are a slew of CHS-effectors involved in the tumor regression. As highlighted before, there is no experimental or mechanistic explanation of a bystander effect, only observational.

The mechanisms of contact hypersensitivity are hard to apply to this context, as the reactions are being induced in a tumor suppressive environment, which may not include many immune cell types [150]. On top of this, the induction of hapten-mediated cell death must be considered, as it likely induces tumor regression and immune responses (Table 2). It is very possible that the tumor regression is due to cell death of all the tumor cells or some combination of cell death and haptenation of the tumor cells. When speculating in this context, it is important to remember that tumor cell death in the tumor can elicit antitumor immune responses, although the type of cell death necessary to mediate immunity remains unclear. As seen in Table 2, it has been shown that in some systems, autophagy from chemotherapy induced the release of HMGB1 and ATP, causing the recruitment and activation of dendritic cells and T-cells [120]. The ATP release may be similar to that seen in CHS, where hapten modification causes ATP release, stimulation of PSRX7 on dendritic cells, and NLRP3 activation. This leads to IL-18 and IL-1*β* release, which can activate dendritic cells in the area. Along with this, haptenation of the tumors may induce the upregulation of CHS chemokines, selectins, and adhesion molecules in the tumor vasculature, causing recruitment of hapten-specific T and NK cells. This could aid in primary tumor regression. Fujiwara et al. [32, 33] used a relatively high concentration of TNCB in large injection volumes, so it is plausible that many of the cancer cells were going to be TNP-bound and died. Low concentrations of haptens induce apoptosis, and higher concentrations, like used in Fujiwara's work, seem to cause necrosis [146, 148]. Hapten-mediated cell death must be considered as a viable mechanism for* in situ* haptenation-induced tumor regression. Theoretically comparing hydrophobic and hydrophilic haptens, such as TNCB and TNBS, respectively, could test this, where TNCB kills bound-cells and TNBS allows further proliferation and growth of bound-cells. A tumor regression experiment using* in situ* haptenation injection with these two haptens (separately) in hapten-sensitized mice would show if it is the TNP haptenation leading to antitumor immune responses, the hapten-mediated cell death that is eliciting tumor regression, or some combination of both.

### 4.6. Epifocal Hapten Application Leading to a CHS-Like Immune Reaction at the Tumor Site

#### 4.6.1. Use of Epifocal Hapten Application to Induce Viral Wart Regression

The contact allergens for topical treatments of various dermatological problems, such as alopecia areata, viral warts, and some cutaneous tumors, have been used since the 1960s. Buckley and Vivier [18] reviewed many of the clinical trials using contact sensitizers to induce viral wart regression. They pointed out that very few of these studies had the proper control groups or randomization, making many of the observations biased and hard to gather conclusions from. The sensitizers mainly used for these trials were DNCB, a potent contact allergen and mutagen first used in 1912, squaric acid dibutyl ester (SADBE), a potent contact allergen first used in 1979, nonmutagenic, and commonly used to treat viral warts in Europe and Southeast Asia, and Diphencyprone (DPCP), a potent contact allergen in humans and animals, nonmutagenic, and commercially available in the UK. All patients given this treatment were usually sensitized under the armpit with ~2% solutions of the hapten. The hapten was then applied to the warts at a concentration of 0.1% (depending on location) and was increased depending on the reaction seen. Application was stopped when there were no visible warts. The mechanism of action for these contact allergens affecting viral warts is not well investigated, although it is theorized that the allergen application induces alterations in cytokine levels, nonspecific inflammation causing wart regression, and haptenation inducing hapten-specific immune responses [18]. It is likely that CHS/ACD-like reactions are occurring in the wart site, although there is little evidence for this. It was seen that CD8+ T-cells infiltrate into warts upon DPCP application, and DNCB application can increase complement-binding wart virus-specific antibodies. Overall, the clearances of warts ranged from 7 to 100% in the trials with a median clearance rate of 62%. It was also seen that long-term, hapten-dependent treatment was needed to cause regression [18].

Upitis and Krol [19] conducted a clinical trial using the hapten diphenylcyclopropenone (DPC) to treat recalcitrant palmoplantar and periungual warts. The study had 154 patients, all of which were sensitized to DPC; 135 of which had complete clearance of warts with an average of 5 treatments over 6 months. There were very few side effects to the treatments, leading the authors to the conclusion that DPC should be considered as a first line treatment for warts. However, the mechanism of action is not well explained. A more recent clinical study [124], treated six facial wart patients, who were not responding to other treatments, with DPCP. Patients were sensitized to 2% DPCP as described above, and various concentrations of DPCP were applied to the warts of interest in 8–10 sessions. Four of six patients had complete disappearance of the warts with no recurrence for a year and the other two patients had improved warts. Once again, the mechanism of action is unknown in this study [124]. Both of these studies seemed to be hapten-dependent phenomena.

Despite the evidence suggesting that contact allergen application can treat warts, warts are known to spontaneously regress and disappear. Many of these studies were over one year, and very frequently, warts will spontaneously regress within a one- to two-year period. Along with this, the mechanism of this viral wart regression remains largely unknown and needs further elucidation, although it is likely that a hapten-dependent CHS-like immune response would have occurred, as most patients were sensitized to the hapten prior to use.

#### 4.6.2. Use of Epifocal Hapten Application to Induce Tumor Regression

Epifocal hapten application at cutaneous tumor sites to elicit CHS-like immune reactions and primary tumor regression is a long-established and appealing concept. Edmund Klein reviewed multiple clinical uses of epifocal hapten application for the treatment of cutaneous cancers [34]. He assessed studies on cutaneous neoplasms, where treatment of epitheliomas using chemotherapy was compared to hapten-induced (2,3,5-triethyleneiminobenzoquinone) [TEIB] and DNCB cutaneous hypersensitivity reactions at the tumor site. These cutaneous hypersensitivity reactions at the tumor site resulted in the regression of superficial basal cell carcinomas (BCC), squamous cell carcinomas (SCC)* in situ*, and premalignant keratosis. In particular, multiple studies on patients with BCC where hypersensitivity was induced by topical application of cream containing 0.05% TEIB were described. A case study was done on one patient receiving this treatment, who had regression of several hundred basal cell carcinomas after 3 weeks of daily topical application. The tumors would become eurythmic, exudated, and necrotic within 24 hours of application. The patient had no recurrence of regressed lesions for 5 years after treatments. Whenever the patient developed new lesions in different sites, the cream was applied and the tumors would disappear. There were also several studies performed on squamous cell carcinoma. The carcinomas* in situ* responded very well to topical challenge with TEIB or DNCB and the reaction was similar to that seen in the basal cell carcinomas. More than 90% of the lesions underwent regression following the hapten challenge, although the deeper lesions responded poorly and did not fully regress, needing secondary treatment with the hapten, chemotherapy, or other standard treatment to eradicate it. These studies clearly demonstrate the powerful ability of haptens to cause CHS reactions in epidermal tumor sites to cause local tumor regression. To note, the hapten-mediated tumor regression did not cause regression of untreated tumors suggesting that hapten-dependent tumor regression was mediated by cell death and/or CHS-like reactions [34].

Epifocal hapten application has been used to topically treat metastatic cutaneous melanoma since 1973. Truchetet et al. [113] reviewed the use of DNCB in the treatment of metastatic melanoma in the clinical settings. Most of these studies used epifocal DNCB application at a concentration of 1–10% in acetone, some using sensitization and some not. In 1978, Loth and Ehring [151] tried the treatment in 35 patients, nine of whom had a favorable response. In 1981, Picrard et al. [152] described 86 cases of primary melanoma with or without metastases treated with DNCB after sensitization. The tumors were excised at multiple time points after treatment. All the patients benefitted from the epifocal applications of DNCB on tumor and normal skin between the primary melanoma and excision of metastases. The 5-year survival was 77% with DNCB application before and after resection versus 70% with DNCB application only after resection. There was no survival benefit seen when the disease had spread to the lymph node. They state that DNCB treatments are only useful for local recurrences and skin metastases, not surgically inaccessible regions. This would imply that the reaction is directly hapten-dependent and a bystander effect is not occurring in a majority of patients as the reactions may be limited to the skin lesions. The mechanism of tumor regression and whether it is mediated by hapten-cell death or CHS like immune reactions was not studied.

Strobbe et al. [35] treated 59 recurrent melanoma patients with a combination of topical DNCB and systemic dacarbazine (DTIC). Patients were sensitized to 2% DNCB on their cutaneous metastasis on day 1 and day 8, followed by additional treatment on day 15. Topical treatments were administered three times per week for 2 weeks. DTIC treatment was started 4 weeks after the first DNCB application with 3 consecutive doses of 400 mg/m^2^, a single dose of 800 mg/m^2^, or 5 consecutive doses of 250 mg/m^2^ and repeated every 3-4 weeks. Of the 59 patients, 15 (25%) had a complete response, 7 (12%) had partial response or stable disease, and 37 (65%) had tumor progression. The overall 5-year survival was 15%, with a median survival of 10 months. The median survival of the group with complete response was 50 months. The presence of severe local reaction to topical DNCB application correlated with improved overall survival. Of the 15 complete responders, 5 patients exhibited a 5-year durable response. Besides these observations, there are no immune correlates reported in this study. This study does not compare the data collected to DTIC only treated patients, which is reported to have a 10.2% response rate in stage IV melanoma patients [153]. DNCB treatment only was also not studied, making it difficult to determine which treatment had an effect. However, they did state that no DNCB-treated lesions disappeared until the start of DTIC treatment. Along with this, they sensitized patients at the tumor site, potentially diminishing the immune reactions as tumors are immune-suppressive. It would have made more impact if the hapten sensitization was given elsewhere as done in many other clinical settings using contact sensitizers to treat metastatic melanoma. Although this study shows a few patients responding to the treatment, the data is not strong enough to suggest a positive response to the treatment.

There have been many case studies using epifocal DNCB or DPCP treatments for melanoma metastases [36, 38–40]. von Nida and Quirk [36] described a patient who was sensitized to 2% DNCB on normal skin and once the appearance of low-grade eczema appeared at that site, the patient was instructed to apply 2% DNCB to the tumor nodules. Within 2 weeks, eczema-like reactions appeared at each site and tumors were all regressing. Tumor nodules continued to appear and regress with treatment for the next 2 years. This went on for 7 years until the patient had liver metastasis and succumbed to the disease. The DNCB treatment in this case seemed to slow the progression of disease by treating cutaneous lesions in a hapten-dependent manner but did not ultimately stop the disease from metastasizing [36]. Damian et al. [39] described seven case studies of metastatic melanoma patients who were sensitized with 2 drops of 2% DPCP in acetone on the upper inner arm for 48 hours. Two weeks after sensitization, DPCP aqueous cream was applied weekly to all cutaneous melanoma metastases. All of them had either slowing of tumor growth or regression of tumors where the DPCP was applied. Three of the patients succumbed to the disease due to metastases within 5 weeks to 19 months, but four were alive at the time of publication. In a follow-up study, the role of Th17 cells in one patient who remained free of cutaneous and regional disease for 4.5 years after DPCP and DTIC treatment of the disease was reported [38]. They observed lymphocyte infiltration into the tumor after treatment marked by “cells [that] display typical morphologic characteristics of melanophages.” However, no specific immunologic stains were performed. RNA expression analysis revealed upregulation of the human Th17 genes (L-17A/B/C/D/E/F; CD27; CD70; PLZF-1; CTLA-4 FoxP3 and ROR*γ*T) in the posttreatment tissue sections. This was not confirmed by looking at the presence of Th17-associated protein or increased Th17 cell infiltration [38]. Lastly, another group [40] reported a patient treated with the same method as Damian et al., [39] which had regression of melanoma nodules on the ankle for up to 18 weeks. This area was dry and eczematous with the appearance of numerous eosinophils (determined by H&E statin, no specific eosinophil markers) and no melanoma (HMB-45 stain).

There was a case report by Herrmann et al. [114] showing complete regression of Merkel cell carcinoma in the scalp 1 year after treatment using a topical DNCB treatment. The patient was sensitized to 2% DNCB and DNCB was applied to the lesions for 4 subsequent weeks. H&E immunostaining of biopsied specimens showed infiltration of CD3+ T-cells and CD28+, KP-1+ Macrophages. To note, mitoses of the tumor cells were still present, but much less frequent than before treatment.

Although these case studies [36, 38–40] suggest a beneficial aspect of the DNCB or DPCP treatment, it is difficult to interpret these results, as case reports are typically the best-case scenario and are from rare patients that have a response. Along with this, it is challenging to compare the study by Strobbe et al. [35] and the case studies [36, 38–40], as Strobbe et al. [35] sensitized patients at the tumor site, which is immune-suppressive and may have dampened sensitization, while the case studies sensitized patients at distant skin sites, allowing for appropriate sensitization. Something that all these studies do show is that the tumor regression seems to be hapten-dependent and seems to not induce a bystander effect, evident from metastases formation. There were very few immune correlations made in any of these studies, only visual observations, making it difficult to interpret how these treatments are inducing tumor regression. It would be interesting to expand the observations by Klein [34] and perform a controlled trial in BCC or SCC patients to establish if this method can indeed induce tumor regression, decrease recurrence of metastatic disease, and potentially increase the patient survival.

Wack et al. [42] created a mouse melanoma model based on Strobbe et al.'s [35] work utilizing DTIC and DNCB and examined the tumor regression mechanisms in B16F17, slow growing B16 substrain, bearing C57BL/6 mice. Seven days after subcutaneous tumor inoculation, when the tumor was 25 *μ*L in volume, mice were treated with i.p. injection of DTIC and/or epifocal (on the skin of the tumor site) DNCB application (25 *μ*L in acetone and olive oil, 4 : 1) 24 hours later. The concentration of both DTIC and DNCB was optimized to be 50 mg/kg DTIC on days 7, 12, 16, and 20 and 3% DNCB on day 8 (to mimic CHS sensitization) and 1% DNCB on days 12, 16, and 20. This treatment regimen resulted in tumor regression and tumor-free mice for up to 150 days in 72% of mice. Lastly, whether or not this treatment would cause tumor regression or resistance of B16F17 lung metastases injected i.v. on day 7 was tested. The combined treatment of DTIC and DNCB was started on Day 9. DTIC and DNCB combination treated mice had significantly less lung metastases than the control and untreated groups 30 days after inoculation. Interestingly, there was no single treatment controls used in many of these experiments, making it difficult to see the effect of the combined treatment compared to the individual treatment effects.

This work has three large issues. (1) The animals were not sensitized to DNCB using normal sensitization procedures. Typically, for CHS reactions, animals are sensitized to the hapten five days before challenge and are sensitized on the distant area (usually the abdomen) from the challenge. This ensures that any reaction being elicited is truly an immune response. The effective sensitization time for DNCB (5 days) was not given and moreover, the sensitization was elicited on the tumor, which is immune suppressive. These two factors probably reduced the sensitization efficacy significantly. The authors mention that they tested the sensitization of different percentages of DNCB using the ear-swelling test, but it is unclear if the DNCB in this setting was applied on the tumor or in a different area of the animal. If the ear-swelling test was performed after sensitization at the tumor site, it would have been prudent to compare the ear swelling to mice sensitized at a nontumor site to see if the sensitization was affected by doing it at the tumor site. (2) All of the tumor measurements here are mean tumor volumes, yet there are no standard deviation or error bars on any of the points. It is difficult to tell what the range of data is and its relative significance. (3) Appropriate controls were not used for each experiment; DTIC treatment alone or DNCB treatment alone was given in the first figure and did not reflect in any subsequent figure. This makes the results difficult to interpret because it is unclear if it is the combination treatment or just a single treatment that caused the observed primary or pulmonary tumor regression.

Wack et al. [41] performed a follow-up study using this model to look into the antitumor immune responses elicited by the DTIC/DNCB combination treatment. Once again, there were no single treatment controls in any of their experiments. They first repeated their previous results showing that 5 of 7 mice underwent complete tumor regression in the 35-day observation period. They looked at the incidence of pulmonary tumors after 7 treatments (the last study used only 4 treatments [42]) and observed that there were only 7 ± 4 tumors in the combination group versus 133 ± 31 in control mice. Splenocytes from treated animals that underwent primary tumor regression were tested for their ability to kill B16-melanoma cells* in vitro* using ^51^Cr-release assay. The cytotoxicity of splenocytes from treated animals toward B16s was 3 times higher than control animals; these splenocytes also released more IFN*γ*. MACS isolated and* in vitro* restimulated CD4+ and CD8+ T-cells each from treated splenocytes had higher killing than the control, whereas the NK cells had similar killing as the control. The similar NK cell killing was expected, as NK cells involved in CHS are derived from the liver [75] and not the spleen. Ability of TILs from the primary B16 tumor to kill B16 melanoma cells and release IFN*γ in vitro* was higher in the treated versus untreated animals. These cells also had high mRNA levels of IFN*γ*, TNF*α*, and IL-6. Using Rag^−/−^ mice, the paper also showed that tumor regression was dependent on T-cells and that this model was repeatable with another hapten, Oxazolone [41].

However, this study also has three large issues. (1) As highlighted before, the single treatment controls were not looked at for any experiment, making it hard to tell if the ability of immune cells to kill or produce cytokines* in vitro* is from the combination of treatments or just one treatment alone. (2) For the cytotoxicity studies using CD4+ T-cells, CD8+ T-cells, and NK cells, they only stimulate these cells with melanoma* in vitro*, not stimulating the cells with DNP-modified melanoma to see if this has an ability to cause cytotoxicity. It is very likely that NK cells will not kill unhaptenated cells because of inhibitory molecules binding to MHC, as previously described. It is hard to draw conclusions from these cytotoxicity assays as stimulation with melanoma and DNP-bound melanoma was not compared. (3) The study used immune cells from the spleen, even though it is commonly known that CHS-related T-cells mature and preside in the draining lymph nodes and CHS-related NK cells reside in the liver. It is very possible that the collected cells had nothing to do with the treatment.

Despite the highlighted issues, these two papers establish the only mouse-model of tumor regression utilizing epifocal hapten application. However, these papers do not elucidate how the tumor regression is being mediated. To further elucidate the validity of this method, these experiments would need to be repeated with all the appropriate single treatment controls taking into consideration the extensive issues present in each paper.

#### 4.6.3. Plausible Immunologic Reactions Linked to Epifocal Hapten Application

When considering the use of epifocal hapten application to induce CHS-like immune reactions at the tumor site, two aspects must be taken into account: (1) haptens will induce cell death and CHS-like immune reactions that may be able to cause tumor regression by utilizing the extensive immune cell milieu (Table 2). (2) Haptens will induce CHS-like immune reactions that may lead to tumor cell growth and increased immune suppression (Table 3).

It is likely that epifocal hapten application induces tumor regression through CHS-like mechanisms (Table 2). First, epifocal hapten application would induce massive cell death in the tumor as any haptenated tumor cell would likely die. In a hapten presensitized animal, tumor haptenation and cell death will cause the release of danger signals, ATP, and ROS. These signals will help induce immune cells in the surrounding tissue. ATP release will induce P2RX7, which will cause the activation of NLRP3 on APCs, eliciting the production of IL-18 and IL-1*β*; these elicit protection against colorectal tumorigenesis by polarizing IFN*γ*+ CD8+ T-cells against tumors in the context of chemotherapy [115]. Release of ROS has the ability to inhibit myeloid derived suppressor cell (MDSC) maturation, known to suppress immune responses against tumors by releasing IL-10 [116], and induce cell death of tumor cells in the established tumor [117]. The stimulation of APCs by danger signals could potentially reactivate exhausted CD8+ T-cells in the tumor microenvironment as DCs are linked to T-cell exhaustion [118, 119] or help APCs traffic to the lymph node to establish new CD8+ effector T-cells. iNKT-cells, activated by CD1d presentation of haptenated tumor glycolipids, and *γδ* T-cells will work together to produce IFN*γ*, which has an antitumor protective role as a potent Th1 cytokine [140] and mediates antitumor activity [150]. iNKT-cell activation will also lead to IL-4 release causing the activation of CS-initiating B-1 cells to produce Hapten-Tumor IgM. This antibody could potentially lead to the coating of cancer cells and subsequent ADCC. This hapten-tumor IgM will also lead to the activation of mast cells which will release TNF*α* and CXCL2, causing cause FasL+, perforin+ neutrophil cell infiltration. These neutrophils may be able to kill the tumor cells in the first 24 hours [121, 122] and provoke release of CXCL1 and CXCL2 from the surrounding tissue, helping T-cells traffic to the tumor site. The mast cells will also release TNF*α* and Serotonin, causing upregulation of chemokines, selectins, and adhesion molecules and subsequent hapten-specific T-cell to trafficking to the tumor. Hapten-specific CD8+ T-cells will enter the area and produce IFN*γ*, which can help to stimulate other effector TILs in the area [125] and cause antitumor activity [150]. Along with this, the entry of hapten-specific CD4+ T-cells could potentially rescue exhausted CD8+ T-cells, as seen in chronic viral infections [123]. The entry of Tc17 and Th17 cells could elicit multiple antitumor immune responses, as CD4+ and CD8+ IL-17 producing T-cells have been shown to elicit tumor regression in melanoma mouse models [126, 127]. Lastly, hapten application could induce the infiltration of CXCR6+ Hepatic NK cells, which may be able to cause tumor cell death once in the site [128]. Despite all the possible reactions that could occur, it is difficult to say if and how these responses would lead to a bystander tumor effect, as there is little evidence for the functionality of hapten-effector cross-reactivity. The only process that could lead to a bystander effect is the massive amount of cell death that occurs from haptenation, causing the release of tumor antigens into the animal and potential immune recognition against these antigens.

There are many aspects of CHS-like reactions that could cause tumor immune suppression and promote tumor cell growth, instead of regression. Bock et al. [154] looked at the ability of continuous DNFB exposure to cause toxicity and tumor formation in multiple different mouse strains. In this study, the animals were exposed to one dose of 7,12-dimethylbenz[*α*]anthracene (DMBA), a known cancer causing agent, and then applied 0.1% DNFB to the site 5 times a week for 14–50 weeks starting 21 days after the DMBA. This caused 35/50 Swiss, 6/30 C57BL/6, and 5/30 Balb/c mice to form tumors. DMBA treatment alone resulted in very low incidence of tumors, 2/50 Swiss, and 0/30 C57BL/6 and Balb/c mice, respectively. There were no tumor formations in Swiss mice (0/50) that were treated with only DNFB. The data suggest that although DNFB is not a causative agent of cancers, it is a tumor-promoting agent and can possibly cause tumor formation in predisposed conditions or already growing tumors with repeated exposure. It is important to note that massive amounts of DNFB were given to these animals over very long periods of time and the mechanism of hapten-mediated tumor promotion was not discussed.

An extensive 24-year study, between 1984 and 2008, by Engkilde et al. [155] looked at the association between contact allergy by small chemicals and cancer incidence. The group patch tested, a way of identifying whether a small molecule causes skin inflammation upon contact, 16,922 patients (6,113 men and 10,809 women), 35.8% of which had a positive reaction to at least one allergen. These results were linked to the Danish Cancer Registry, where the group saw that 3,200 (18.9%) of the dermatitis patients had some type of cancer and that 1,207 (37.7%) of these patients had a positive patch test. The group found significant correlations between contact allergy and bladder, breast, and skin (nonmelanoma) cancer regardless of sex. There was also an inverse correlation between a positive patch test and brain/CNS cancer in women. This study underscores that the reactions causing ACD, like those involved in CHS, may be associated with cancer in certain cases.

We have conceptualized some of the possible mechanisms of hapten-induced CHS promoting tumor immune suppression and tumor growth (Table 3). Epifocal application of a hapten will cause the release of danger signals, such as PGE2, ROS, and ATP. PGE2 release has been seen to induce colon cancer growth [137] and cause MDSC activation in the tumor site. ROS release is known to upregulate VEGF, promoting angiogenesis in tumor sites [138], and possibly cause the nitration of T-cell-peptide-MHC interactions, inducing T-cell suppression [116]. ATP release will induce P2RX7, which will cause the activation of NLRP3 on dermal APCs, eliciting the production of IL-1*β* and IL-18 which has been shown to decrease the tumor responsiveness to certain vaccinations [115]. The danger signal release will cause TLR4 and TLR2 stimulation of dermal APCs, which has been shown to elicit immune evasion by helping myeloid cells establish metastases via TGF-*β* [115, 116]. Haptenation will also cause keratinocytes to release IL-1*β*, IL-6, IL-18, and TNF*α*, which have been shown to cause MDSC recruitment and infiltration at the tumor site, subsequently causing IL-10 release and immune suppression [116]. Keratinocytes will also cause CXCL10 upregulation, which has been shown to elicit angiogenesis [139]. iNKT-cell activation will cause release of IL-4 and IL-13, which are both known to elicit MDSC recruitment and infiltration [116] as well as direct suppression of tumor-specific CD8+ T-cells [140]. Mast cell activation by complement C5a will cause CCL2 and CCL5 upregulation, which has been to shown to induce Tumor Associated Macrophages (TAMs) to release IL-10, promote angiogenesis, and stimulate tumor metastasis [116]. Mast cells will also release TNF*α*, known to help deliver oxygen to hypoxic areas of the tumor allowing for tumor growth [116], and release CXCL2, seen to induce melanoma cell proliferation [139]. Lastly, the induction of CHS at the tumor site could cause the infiltration of hapten-specific T-regs, which could potentially release IL-10 to suppress effector T-cells [141] or elicit CD8+ T-cell exhaustion by expression of CTLA-4 [118].

It is likely that the antitumor immunity or tumor-mediated immune suppression and tumor growth due to elicitation of CHS from epifocal hapten application will have much to do with the (a) type of tumor treated (b) growth rate of the tumor, and (c) timing of the administration. It is suggested, by hapten-specific T-cell migration data, that no antigen presentation occurs outside of the dermis in the CHS elicitation phase [86]. This finding makes it likely that epifocal hapten application will only be useful for treating cutaneous cancer. The mechanisms of hapten-induced tumor regression using epifocal hapten application still remain unclear and need to be further studied. It is also essential to figure out the situations in which a hapten will induce tumor regression versus tumor growth by testing several different haptens in well-defined systems, which have yet to be created. If all this is done, it can be understood if epifocal hapten application is useful in eliciting tumor regression and antitumor immune responses.

### 4.7. Antigen-Hapten Conjugate-Mediated Antibody-Dependent Cellular Cytotoxicity

From 2002 to 2013, Philip S. Low's group used a unique approach to hapten-mediated tumor treatment. They synthesized folate-hapten conjugates and used them to treat folate receptor high cancers. The concept is that the folate would bind to folate receptors on the tumors coating the tumors in haptens, which could lead to ADCC and complement system activation, effectively killing the tumor in hapten-sensitized animals. In their work, they utilized the haptens FITC and DNP, and treated folate high M109 lung carcinomas. This treatment is not directly cytotoxic like direct haptenation. It is important to note that the immune mechanisms occurring here are wildly different than what has been described earlier (Sections  1, 2, and 3 of the hapten-mediated tumor treatments) having little to do with CHS mechanisms, and mostly mediated by hapten-induced ADCC. These studies present a good mechanistic view of how the tumor regression is occurring.

Lu and Low [46] conjugated the Th2-hapten FITC to folate [46]. They treated cancer cells* in vitro* with the Folate-FITC conjugates, ensuring the FITC coating of M109 cells. Balb/c mice were inoculated with M109 cells and sensitized to BSA-FITC, inducing a strong anti-FITC antibody response. Intravenous injection of Folate-FITC coated s.c. M109 tumors within one day. They observed slight increase in survival in mice with peritoneal M109 tumors with the IL-2 or Folate-FITC alone (i.p. administration), but large increase in survival with the combination of the therapies. They added IFN*α* treatment to the IL-2 + Folate-FITC, which showed a very significant increase in survival, from a maximum of 30 days up to over 80 days in 20% of the animals. After immense optimization of folate-FITC, IL-2, and IFN*α* concentrations, they were able to find a curative treatment that gave 100% survival of mice for 100 days. They rechallenged long-term survivors with the same number followed by 3x as many M109 cells and saw that the mice were able to survive the rechallenges, suggesting long-term immunity in these mice; this was only shown as survival curves, so it was unclear if the tumors grew or not. Of note, many cells in the body express the folate receptor and this treatment could cause FITC coating and ADCC at distant, folate receptor expressing sites [156]. Realizing this, the authors submitted cured animals for toxicological analysis where it was determined that the treatment was not toxic and that there was no opsonization or damage of organs [46]. Along with that, IL-2 and IFN*α* treatments are known to cause side effects in clinical use, so combining them with the folate-FITC conjugate could increase any potential side effects [157]. Despite these worries, they clearly showed that this method coated tumors cells* in vivo* with FITC and significantly increased mouse survival in combination with cytokine treatment.

Lu et al., [45] then studied the immune mechanisms of folate-FITC-mediated tumor regression. They observed a bimodal plot of folate-FITC at various concentrations; this is commonly seen in treatments that do not directly kill tumor cell. There was no complement-mediated lysis of folate-FITC-labeled tumor cells occurring. NK cells showed direct lysis of folate-FITC coated tumor cells in the presence of anti-FITC antibody, suggesting ADCC. Macrophages engulfed the folate-FITC-bound tumor cells opsonized with FITC antiserum and ~34% of these cells were engulfed after a 30-minute coculture. These data suggest that both NK cells and macrophages are involved in killing and clearing folate-FITC/anti-FITC antibody marked tumor cells. Using the complete treatment* in vivo*, they compared the survival of treated control mice and NK cell-depleted mice, showing a decrease in the overall survival, back to the basal level without NK cells. Depletion of CD4+ T-cells and CD8+ T-cells alone and in combination and depletion of macrophages significantly decreased the overall survival of the mice, close to that of the untreated mice, but not as extreme a decrease as the NK cell depletion. CD8+ T-cells were removed from cured animals and were seen to kill M109 cells better than T-cells from untreated animals, suggesting that this treatment is eliciting T-cell memory against the tumor. However, they did not perform adoptive transfer experiments to see if these cells could clear M109 tumors in naïve animals. Lastly, they showed that the optimized treatment was able to fully regress the tumor for 35 days, whereas the controls (PBS and PBS + IL-2/IFN*α*) had little effect.

These papers provide strong evidence for folate-FITC-mediated tumor regression and underlying immune mechanisms of this regression. However, it must be determined what the role of CD4+ and CD8+ T-cells is in this treatment and how the animals are clearing secondary tumor challenges. It is likely that macrophages are presenting tumor antigens after opsonization, causing the formation of tumor-specific T-cells. This is likely the reason CD4+ and CD8+ T-cells are important for animal survival.

Lu et al. [44] performed preclinical pharmacokinetics and tissue distribution studies. They utilized a radioactive folate-FITC conjugate to track the movement of the conjugate* in vivo* and saw that it was rapidly eliminated in naïve mice but formed immune complexes with FITC-specific antibodies in FITC sensitized animals, causing an extended duration of folate-FITC in the animal (173-fold increase in drug exposure). Extremely high doses of the folate-FITC were shown to cosaturate the tumor cell's folate receptors and the circulating FITC-specific antibodies, hindering immune recognition of the tumor and thereby lowering the antitumor activity.

Lu et al. [43] also established folate-DNP conjugates (EC57, EC63, EC0293, and EC0294) that showed similar results to the folate-FITC conjugate when using similar treatment regimens. One (EC0294) of four tested-conjugates, in combination with IL-2 and IFN*α*, markedly improved survival of M109 tumor bearing mice for more than 100 days; two of the treatments, EC0293 and EC0294, gave 40 and 60% cure rates, respectively, among these mice. They did not include tumor regression data. The cured mice all rejected the secondary tumor inoculation of M109 cells, suggesting an antitumor immune response. They looked into the risk of an allergic response, passive cutaneous anaphylaxis assay, to the treatment and saw that the conjugates that gave allergic responses were the ones that cured mice. These results show that the folate-DNP conjugates can elicit prolonged survival, secondary tumor rejection, and autoimmune side effects; however, they do not show direct tumor regression results. This study shows that the concept of antigen-hapten treatment is a very effective treatment for folate receptor high cancers as it can be done with different haptens (FITC and DNP) and potentially elicits long-term tumor immunity. It would be interesting to know if other antigen-receptor targets could elicit similar results.

Recently, Low's group [47] published a phase I clinical study using the folate-FITC treatment alone in patients with renal cell carcinoma. Patients were given EC90, the hapten fluorescein, with the adjuvant GPI-0100 to stimulate the production of anti-FITC antibodies followed by EC17, the folate-FITC conjugate treatment. 39 patients got at least one dose of the EC90, and 33 received at least one dose of the EC17 treatment. Of the 33 patients that received the EC17 treatment, 28 patients had baseline and at least one had follow-up tumor assessment. Of 28 patients, 1 (4%) patient achieved partial response, 15 (54%) patients achieved stable disease, and 12 (43%) had progressive disease. Of the 16 patients that completed 2 cycles of the EC17 therapy, 12 (75%) had stable disease and 4 (25%) had progressive disease and of the 11 patients that completed 3 cycles of the therapy, 6 (55%) had stable disease and 5 (45%) had progressive disease. There was no apparent relationship found between the anti-FITC antibody titer and the best response to the therapy. Although many patients had stable disease, only one had partial regression and no patients had complete regression.

These results are not unexpected, as the mouse treatment required the use of IL-2 and IFN*α* treatments to be fully effective. In the clinical study, patients were also not sensitized to the hapten, likely affecting the results. This trial was likely performed to see the side effects of the folate-FITC conjugate alone on patients. As stated in the phase I study, Low's group has completed a phase II trial of the EC17 treatment in combination with cytokine treatment and we hope those results will be published soon. It still needs to be determined how tumor challenges are rejected using this method.

## 5. Conclusions

Evidently, the field of contact hypersensitivity is still expanding, as there are many conflicting reports on several different aspects of the mechanism. The use of different mouse strains, different haptens, and different administrations or concentrations of haptens greatly impacts the immune responses seen. It would be paramount to attempt to standardize the methods of inducing CHS, so that more clear mechanisms can be established between different haptens and mouse strains. There is much work to be done to fill in the gaps and confirm parts of the pathway that remain unclear. Obviously, the use of haptens and haptenation as a tumor treatment needs further research to determine its efficacy. Much of the work with hapten-inducing tumor regression was done before the field of CHS was developed to its present state, and without in-depth immunologic mechanism depiction. This leaves much speculation about all the results found, as we underscored in this review.

Of the four concepts, antigen-hapten delivery seems to be the most appealing, but it uses completely different tumor clearance than the other treatment mechanisms, as it is mediated by ADCC. The work done by Low's group [43–47] is detailed in explaining the tumor regression mechanism; however, further research is needed to understand if the treatment of folate-FITC along with IL-2 and IFN*α* can be effective. Along with this, it must be understood how tumor rechallenges are rejected after treatment.

For the field of hapten-mediated tumor regression to move forward, we propose that each model of hapten-mediated tumor regression be fully studied so that the mechanisms of primary and secondary tumor regression become clear. In this regard, we urge that the field must also consider the effect of hapten-mediated cell death, as the dead cells, like irradiated cells, may elicit antitumor immunity; it needs to be determined if hapten modification alone (on the surface) or hapten modification followed by cell death is needed to mediate antitumor immune responses. It also must be determined whether or not hapten-induced tumor regression can induce bystander effects or if it is hapten-dependent.

Lastly, it is very important to realize that no hapten treatment has been effective without the combination of another immune- or tumor-modulating agent(s), suggesting that haptens may never be able to elicit complete tumor regression by themselves. If this is true, haptens may be considered as adjuvants to possibly increase tumor regression and antitumor immunity by combining them with other tumor treatments that have measurable efficacy. Much of the data on hapten-mediated tumor treatments is observational; thus more mechanistic studies using similar mouse models and haptens as well as more stringently-controlled clinical trials are essential to determine if haptens are appropriate as cancer immunotherapies.

## Figures and Tables

**Table 1:** Summary of the hapten-mediated tumor regression studies.

Hapten treatment	Author, year	Hapten used for treatment, alone and in combination	Tumor type/cell line used in animal and human studies	Route of administration of haptens and hapten-modified products	Observations
*Ex vivo* haptenation	Hamaoka et al., 1979 [21]	TNBS, TNP-MGG sensitization and TNP-D-GL pretreatment	X5563 cells in C3H/HeN mice	i.p. TNP-X5563 injection	Significantly delayed tumor growth for up to 15 days
	Fujiwara et al., 1980 [22]	TNBS, TNP-MGG sensitization and TNP-D-GL pretreatment	LSTRA cells in Balb/c mice	i.p. TNP-X5563 injection	Significantly delayed tumor growth for up to 10 days
	Flood et al., 1987 [23]	TNBS, N/A	Progressor and regressor fibrosarcomas in C3H/HeN mice	s.c. TNP-regressor/TNP-progressor injection	Significantly delayed tumor growth for up to 30 days
	Berd et al., 1993 [30]	DNFB, DNFB sensitization and CY pretreatment combined with BCG and nodal resection	Stages III and IV metastatic melanoma in patients	i.d. DNP-autologous melanoma injection	5/46 patient responses for metastatic melanoma and 59% 2-year survival postnodal resection
	Sato et al., 1995 [29]	DNFB, DNFB sensitization and CY pretreatment combined with BCG and nodal resection	Stages III and IV metastatic melanoma in patients	i.d. DNP-autologous melanoma injection	IFN*γ* producing CD8 T cells that killed DNP-melanoma only
	Sato et al., 1997 [27]	DNFB, DNFB sensitization and CY pretreatment combined with BCG and nodal resection	Stages III and IV metastatic melanoma in patients	i.d. DNP-autologous melanoma injection	DNP-specific T-cells recognize only hapten-modified melanoma
	Berd et al., 1997 [28]	DNFB, DNFB sensitization and CY pretreatment combined with BCG and nodal resection	Stage III metastatic melanoma postnodal resection in patients	i.d. DNP-autologous melanoma injection	5-year 45% relapse-free and 58% overall survival (62 patients)
	Berd et al., 2001 [26]	DNFB, DNFB sensitization and CY pretreatment combined with BCG and nodal resection	Stage IV melanoma with pulmonary metastases in patients	i.d. DNP-autologous melanoma injection	11/83 patients had responses to treatment, only 2 had complete response
	Manne et al., 2002 [25]	DNFB, DNFB sensitization and CY pretreatment combined with BCG and nodal resection	Stage III metastatic melanoma postnodal resection in patients	i.d. DNP-autologous melanoma injection	T-cell clones from DNP-vaccine patients with similar TCR VDJ peaks and CDR3 amino acid sequences
	Sojka et al., 2002 [31]	DNFB, CY pretreatment combined with BCG and nodal resection	410.1 cells in Balb/c mice	s.c. DNP-410.1 injection	40% relapse-free survival with DNP-vaccine versus 20% without DNP; CD4+, and CD8+ T cells, and IFN*γ* and TNF*α* important for survival.
	Berd et al., 2004 [24]	DNFB, DNFB sensitization and CY pretreatment combined with BCG and nodal resection	Stage III metastatic melanoma postnodal resection in patients	i.d. DNP-autologous melanoma injection	5-year 44% overall survival (214 patients)

*In situ* haptenation	Fujiwara et al., 1984 [32]	TNCB, TNCB sensitization and CY pretreatment	X5563 cells in C3H/HeN mice	Intratumoral injection of TNCB	>50% primary tumor regression and secondary tumor resistance. Helper T-cells crucial
	Fujiwara et al., 1984 [33]	TNCB, TNCB sensitization and CY pretreatment	X5563 cells, MCH-1-A1 cells, and MCA-induced tumors in C3H/HeN mice	Intratumoral injection of TNCB	>50% primary tumor regression and secondary tumor resistance. Helper T-cells crucial

Epifocal hapten application	Klein 1969 [34]	TEIB and DNCB, N/A	BCC and SCC in patients	Topical hapten application on tumor	Reviews various complete tumor regression cases in various different cancers and patients.
	Truchetet et al., 1989 [113]	DNCB, N/A	Metastatic melanoma in patients	Topical DNCB application on tumor	Reviews the use of DNCB to treat metastatic melanoma in the clinic and in case studies
	Strobbe et al., 1997 [35]	DNCB, DNCB sensitization on tumor and systemic DTIC	Recurrent melanoma in patients	Topical DNCB application on tumor	25% complete response with combined DNCB and DTIC treatment
	von Nida and Quirk, 2003 [36]	DNCB, DNCB sensitization	Metastatic melanoma in patients	Topical DNCB application on tumor	Tumor control for 7 years in metastatic melanoma patient with DNCB application
	Herrmann et al., 2004 [114]	DNCB, DNCB sensitization	Merkel cell carcinoma in patients	Topical DNCB application on tumor	Complete tumor regression on scalp and CD3+ T-cell and CD28+, KP-1+ Macrophage infiltration
	Damian et al., 2009 [39]	DPCP, DPCP sensitization	Metastatic melanoma in patients	Topical DPCP application on tumor	Of 7 patients, many had slow growing tumors or tumor regression at DPCP application site
	Martiniuk et al., 2010 [38]	DPCP, DPCP sensitization	Metastatic melanoma in patients	Topical DPCP application on tumor	Role of Th17 cells in tumor regression
	Kim 2012 [40]	DPCP, DPCP sensitization	Metastatic melanoma in patients	Topical DPCP application on tumor	Regression of melanoma nodules for 18 weeks
	Wack et al., 2001 [42]	DNCB, DNCB sensitization on tumor and systemic DTIC	B16F17 cells in C57BL/6 mice	Topical DNCB application on tumor	72% primary tumor regression and reduced pulmonary metastases
	Wack et al., 2002 [41]	DNCB, DNCB sensitization on tumor and systemic DTIC	B16F17 cells in C57BL/6 mice	Topical DNCB application on tumor	Repeat 2001 results, CD4+ and CD8+ T cells kill B16 *in vitro* and release IFN*γ*

	Lu and Low 2002 [46]	Folate-FITC conjugate, BSA-FITC sensitization with adjuvant GPI-0100 and systemic IL-2 and IFN*α*	M109 cells in Balb/c mice	i.v. and i.p. injection folate-FITC conjugate	FITC coating of tumors. 100% overall survival after optimization with combined treatment; survive secondary challenges
	Lu et al., 2005 [45]	Folate-FITC conjugate, BSA-FITC sensitization with adjuvant GPI-0100 and systemic IL-2 and IFN*α*	M109 cells in Balb/c mice	i.p. injection folate-FITC conjugate	NK-cell induced ADCC and Macrophage opsonization; CD4+ and CD8+ T-cells important. Complete tumor regression in 35 days
Antigen-hapten administration	Lu et al., 2006 [44]	Folate-FITC conjugate, BSA-FITC sensitization with adjuvant GPI-0100 and systemic IL-2 and IFN*α*	M109 cells in Balb/c mice	i.p. injection folate-FITC conjugate	Preclinical pharmacokinetic and tissue distribution studies
	Lu et al., 2007 [43]	Folate-DNP conjugate, KLH-DNP sensitization with adjuvant GPI-0100 and systemic IL-2 and IFN*α*	M109 cells in Balb/c mice	i.p. injection folate-DNP conjugate	60% cure-rate in mice
	Amato et al., 2013 [47]	EC17 folate-FITC conjugate, EC90 hapten fluorescein with adjuvant GPI-0100	Renal cell carcinoma in patients	s.c. injection folate-FITC conjugate	Phase-1 Study, 1/28 patients had partial response, 15/28 had stable disease; side effects

**Table 2:** Contact hypersensitivity immune mechanisms that may lead to tumor regression.

CHS immune cell	CHS immune reaction	Plausible direct and indirect mechanisms of tumor regression
Hapten modification of epidermal cells → release of danger signals	ATP release → P2RX7 → NLRP3 activation	IL-18 and IL-1*β* → protection against colorectal tumorigenesis [115]
	ROS	Inhibit MDSC maturation [116]
		Induce cell death in established tumor [117]

Dermal APCs	Stimulation by haptenization	Possibly stimulate exhausted CD8+ T-cells [118, 119]

Keratinocytes	IL-18 release	Protection against colorectal tumorigenesis [116]
	IL-1*β* release	Polarization of IFN*γ* CD8+ T-cells [115]

iNKT cells	IFN*γ* production	Protective role dependent on Th1 cytokines [140] and antitumor activity [150]

Mast cells	TNF*α* and CXCL2 release	Neutrophil activation [4]
	TNF*α* and serotonin release	Chemokine, selectin and adhesion molecule upregulation for hapten-specific T-cell trafficking

Neutrophils	KC damage (FasL and perforin)	Potential tumor damage, although neutrophils not known to directly kill tumor cells in the first 24 hours [121, 122]
	CXCL1 and CXCL2	Chemokine, selectin and adhesion molecule upregulation for hapten-specific T-cell trafficking

CS initiating B-1 cells	Hapten-antibody production	Hapten-tumor IgM → ADCC

CD8+ T-cells	IFN*γ*	TIL activation [125] and antitumor activity [150]
	Hapten-specific CD8+ T-cells	Haptenated-tumor cell killing
	Infiltration into CHS site	Tumor-infiltrating lymphocytes [125]

CD4+ T-cells	Hapten-specific	Rescue exhausted CD8+ T-cells [123]

Tc17/Th17	IL-17 CD4+ and CD8+ Cells	Antitumor immune responses [126, 127]

Hepatic NK cells	Hapten-specific NK-cells	Hapten-tumor cell killing [128]

→: Leads to …

**Table 3:** Contact hypersensitivity reactions that may lead to tumor growth.

CHS immune cell	CHS immune reaction	Plausible direct effect on tumor	Plausible immune suppression that may lead to tumor growth
Hapten modification of epidermal cells → release of danger signals	Prostaglandin E2 (PGE2) release	Colon cancer growth [137]	MDSCs activation [116]
	ROS release	Angiogenesis through VEGF [138]	Nitration of T-cell-peptide-MHC interaction → T-cell suppression [116]
	ATP release → P2RX7 → NLRP3 activation	N/A	Decreased tumor responsiveness to vaccination [115]

LCs and dDCs	TLR4 and 2 Stimulation	N/A	Immune evasion and myeloid cells to promote metastases [115, 116]

Keratinocytes	IL-1*β*, IL-6, IL-18, and TNF*α*	N/A	MDSCs recruitment and infiltration → IL-10 production in tumor site [116]
	CXCL10 Upregulation	Angiogenesis [139]	N/A

iNKT cells	IL-4 and IL-13	N/A	MDSCs and M2MΦ recruitment and infiltration → IL-10 and TGF*β* production in tumor site [116]; Suppression of tumor-specific CD8+ T-cells [140]

Mast cells	CCL2 and CCL5 upregulation	N/A	TAMs (IL-10 high, IL-12 low, IL-1R*α* high, and IL-1decoyR high) → IL-10, angiogenesis, tumor metastasis stimulation, TGF*β*, TNF*α*, IL-1*α* [116]; MDSCs recruitment and infiltration → IL-10 production in tumor site [116]
	TNF*α*	Oxygen delivery to hypoxic tumor cells [116]	N/A
	CXCL2	Melanoma cell proliferation [139]	N/A

Neutrophils	CXCL1 and CXCL2	Melanoma cell proliferation [116, 139]	N/A

Hapten-specific T-regs	IL-10	N/A	Effector T-cell suppression [141]
	CTLA-4	N/A	CD8+ T-cell exhaustion [118]

→: Leads to …
